# Supplementary material for: The Interactions between Two Fungal Endophytes Epicoccum layuense R2-21 and Alternaria alternata XHYN2 and Grapevines (Vitis vinifera) with De Novo Established Symbionts under Aseptic Conditions
Source: J Fungi (Basel). 2023 Nov 30;9(12):1154. doi: 10.3390/jof9121154 (PMC10744766; doi:10.3390/jof9121154)
Supplement: Supplementary file 1 [file jof-09-01154-s001.zip › Tables S1-S8.pdf]

**Table S1.** Primer sequences used in real-time PCR.

| **Gene ID** | | **Sequences (5'-3')** |
|---|---|---|
| *VIT_16s0039g01300* | F | GAGCAAAGGCACCGACAG |
| | R | TCCGTTCCCAAATATCCC |
| *VIT_14s0060g02320* | F | GCCACAAGACGAGCCTAC |
| | R | CCCGAGTCCCTGAAACTA |
| *VIT_16s0100g00840* | F | ATGGACGAGATGAGAAAG |
| | R | CTAGGAATACTGTGGAGGA |
| *VIT_11s0065g00350* | F | CTGTAGCAGGTGGGAAGG |
| | R | TATGCGGTGATTGGAGTG |
| *VIT_10s0042g00870* | F | GCAGCCTAAGTCCAAGAT |
| | R | GTTTCAGAAGGTCCACGA |
| *VIT_05s0094g00200* | F | TCGTATCCTCGGTTTGGC |
| | R | GCATCGGTTGCTACTATTTC |
| *EF1* | F | AGACCACCAAATACTACTGC |
| | R | GATCATCTGCCTGACACC |

Note: F and R represent the upstream and downstream primer, respectively.

**Table S2.** Summary of RNA-seq data from the tissue cultured seedlings with fungal endophytes Epi R2-21 and Alt XHYN2 inoculation. R2-21_6h, R2-21_6d, and R2-21_15d represent leaf samples from Epi R2-21 treatments collected at 6 h, 6 d, and 15 d after inoculation, respectively; XHYN2_6h, XHYN2_6d, and XHYN2_15d represent leaf samples from Alt XHYN2 treatments collected at 6 h, 6 d, and 15 d after inoculation, respectively; and Con_6h, Con_6d, and Con_15d represent leaf samples from the control collected at 6 h, 6 d, and 15 d after inoculation, respectively.

| Samples | Raw reads | Clean reads | Clean Bases | Error% | GC% | Q20% | Q30% | Mapped Reads | Multiple Mapped | Uniquely Mapped | Exons | Introns | Intergenic |
|---|---|---|---|---|---|---|---|---|---|---|---|---|---|
| Con_6h_1 | 51,357,786 | 51,025,210 | 7,620,542,469 | 0.02 | 46.03 | 98.84 | 96.17 | 47,320,467 (92.74%) | 1,314,858(2.58%) | 46,005,609(90.16%) | 94.20% | 3.98% | 1.82% |
| Con_6h_2 | 61,261,982 | 60,785,786 | 9,085,488,738 | 0.02 | 46.14 | 98.79 | 96.00 | 56,375,618 (92.74%) | 1,629,527(2.68%) | 54,746,091(90.06%) | 94.36% | 3.82% | 1.82% |
| Con_6h_3 | 53,306,688 | 52,952,242 | 7,910,426,345 | 0.02 | 46.12 | 98.77 | 95.93 | 49,181,483 (92.88%) | 1,475,902(2.79%) | 47,705,581(90.09%) | 94.58% | 3.72% | 1.71% |
| Con_6d_1 | 51,712,868 | 51,367,466 | 7,666,966,842 | 0.02 | 46.19 | 98.75 | 95.91 | 47,454,315 (92.38%) | 1,215,812(2.37%) | 46,238,503(90.02%) | 93.59% | 4.67% | 1.75% |
| Con_6d_2 | 51,197,876 | 50,807,504 | 7,578,701,758 | 0.02 | 46.15 | 98.71 | 95.84 | 47,017,830 (92.54%) | 1,268,323(2.5%) | 45,749,507(90.04%) | 93.47% | 4.76% | 1.77% |
| Con_6d_3 | 59,676,928 | 59,265,988 | 8,845,307,094 | 0.02 | 46.30 | 98.79 | 96.03 | 54,859,451 (92.56%) | 1,437,521(2.43%) | 53,421,930(90.14%) | 93.90% | 4.40% | 1.70% |
| Con_15d_1 | 55,143,516 | 54,683,956 | 8,158,046,522 | 0.02 | 46.12 | 98.68 | 95.74 | 50,132,738 (91.68%) | 1,203,666(2.2%) | 48,929,072(89.48%) | 92.54% | 5.52% | 1.94% |
| Con_15d_2 | 55,013,022 | 54,617,058 | 8,149,656,904 | 0.02 | 45.96 | 98.77 | 95.99 | 50,579,749 (92.61%) | 1,258,236(2.3%) | 49,321,513(90.3%) | 92.88% | 5.38% | 1.73% |
| Con_15d_3 | 64,744,400 | 64,145,148 | 9,568,177,306 | 0.02 | 46.30 | 98.78 | 96.03 | 59,637,905 (92.97%) | 1,534,916(2.39%) | 58,102,989(90.58%) | 93.81% | 4.56% | 1.63% |
| R2-21_6h_1 | 57,158,658 | 56,754,514 | 8,457,173,641 | 0.02 | 46.16 | 98.80 | 96.05 | 52,939,728 (93.28%) | 1,708,600(3.01%) | 51,231,128(90.27%) | 94.88% | 3.45% | 1.66% |
| R2-21_6h_2 | 66,604,334 | 66,089,034 | 9,859,712,045 | 0.02 | 46.16 | 98.82 | 96.12 | 61,736,428 (93.41%) | 1,921,213(2.91%) | 59,815,215(90.51%) | 95.02% | 3.38% | 1.60% |
| R2-21_6h_3 | 51,582,814 | 51,239,252 | 7652963100 | 0.02 | 46.10 | 98.76 | 95.91 | 47,776,573 (93.24%) | 1,448,960(2.83%) | 46,327,613(90.41%) | 94.62% | 3.72% | 1.67% |
| R2-21_6d_1 | 54,024,656 | 53,596,880 | 8,006,360,201 | 0.02 | 46.30 | 98.75 | 95.95 | 49,381,290 (92.13%) | 1,608,112(3.0%) | 47,773,178(89.13%) | 94.38% | 3.90% | 1.72% |
| R2-21_6d_2 | 60,737,502 | 60,323,284 | 9,003,025,347 | 0.02 | 46.35 | 98.80 | 96.08 | 55,454,165 (91.93%) | 1,756,218(2.91%) | 53,697,947(89.02%) | 94.28% | 3.91% | 1.80% |
| R2-21_6d_3 | 65,150,142 | 64,731,830 | 9,671,180,821 | 0.02 | 46.16 | 98.82 | 96.10 | 59,614,638 (92.09%) | 1,823,454(2.82%) | 57,791,184(89.28%) | 93.89% | 4.30% | 1.80% |
| R2-21_15d_1 | 59,399,898 | 58,901,378 | 8,808,566,614 | 0.02 | 46.26 | 98.75 | 95.93 | 54,153,789 (91.94%) | 2,622,113(4.45%) | 51,531,676(87.49%) | 94.58% | 3.75% | 1.67% |
| R2-21_15d_2 | 60,753,388 | 60,333,808 | 9,000,694,780 | 0.02 | 46.39 | 98.82 | 96.11 | 55,579,956 (92.12%) | 1,973,024(3.27%) | 53,606,932(88.85%) | 94.58% | 3.82% | 1.60% |
| R2-21_15d_3 | 52,121,686 | 51,698,858 | 7,716,249,914 | 0.02 | 46.50 | 98.72 | 95.91 | 47,504,328 (91.89%) | 1,579,696(3.06%) | 45,924,632(88.83%) | 94.47% | 3.94% | 1.58% |

| | | | | | | | | | | | | | |
|---|---|---|---|---|---|---|---|---|---|---|---|---|---|
| XHYN2_6h_1 | 60,480,270 | 60,064,480 | 8,964,140,373 | 0.02 | 46.37 | 98.75 | 95.88 | 55,644,290 (92.64%) | 2,072,563(3.45%) | 53,571,727(89.19%) | 94.91% | 3.37% | 1.72% |
| XHYN2_6h_2 | 58,680,604 | 58,302,532 | 8,688,498,601 | 0.02 | 46.29 | 98.81 | 96.06 | 54,436,119 (93.37%) | 1,838,836(3.15%) | 52,597,283(90.21%) | 95.23% | 3.19% | 1.58% |
| XHYN2_6h_3 | 59,516,776 | 59,128,734 | 8,821,960,312 | 0.02 | 46.24 | 98.81 | 96.06 | 55,229,875 (93.41%) | 1,903,490(3.22%) | 53,326,385(90.19%) | 95.25% | 3.19% | 1.55% |
| XHYN2_6d_1 | 53,705,882 | 53,281,838 | 7,950,209,687 | 0.02 | 46.70 | 98.72 | 95.86 | 48,224,940 (90.51%) | 1,561,463(2.93%) | 46,663,477(87.58%) | 93.89% | 4.07% | 2.05% |
| XHYN2_6d_2 | 51,996,724 | 51,650,776 | 7,710,081,219 | 0.02 | 46.09 | 98.83 | 96.14 | 47,655,267 (92.26%) | 1,491,626(2.89%) | 46,163,641(89.38%) | 93.77% | 4.47% | 1.76% |
| XHYN2_6d_3 | 56,696,930 | 56,252,384 | 8,394,422,963 | 0.02 | 46.27 | 98.73 | 95.90 | 51,885,796 (92.24%) | 1,617,533(2.88%) | 50,268,263(89.36%) | 94.43% | 3.92% | 1.66% |
| XHYN2_15d_1 | 54,268,496 | 53,837,312 | 7,985,326,127 | 0.02 | 46.36 | 98.83 | 96.16 | 49,533,305 (92.01%) | 1,767,125(3.28%) | 47,766,180(88.72%) | 94.64% | 3.70% | 1.65% |
| XHYN2_15d_2 | 53,308,960 | 52,924,606 | 7,909,373,565 | 0.02 | 46.52 | 98.79 | 96.03 | 47,956,750 (90.61%) | 2,907,848(5.49%) | 45,048,902(85.12%) | 94.93% | 3.52% | 1.56% |
| XHYN2_15d_3 | 53,426,418 | 53,052,574 | 7,917,360,000 | 0.02 | 46.45 | 98.81 | 96.09 | 48,553,473 (91.52%) | 2,272,331(4.28%) | 46,281,142(87.24%) | 94.47% | 3.88% | 1.65% |

**Table S3.** DEGs involved in the phenylpropanoid biosynthesis pathway of the tissue cultured seedlings in response to endophytes fungi Epi R2-21 and Alt XHYN2 inoculation ($|Log_2FC|\geq1$; *p*-adjust <0.05). DEGs were analyzed in six pairwise comparisons, R2-21_6h vs. Con_6h comparison, R2-21_6d vs. Con_6d, R2-21_15d vs. Con_15d, XHYN2_6h vs. Con_6h, XHYN2_6d vs. Con_6d, XHYN2_15d vs. Con_15d. R2-21_6h, R2-21_6d, and R2-21 15d represent leaf samples from Epi R2-21 treatments collected at 6 h, 6 d, and 15 d after inoculation, respectively; XHYN2_6h, XHYN2_6d, and XHYN2_15d represent leaf samples from Alt XHYN2 treatments collected at 6 h, 6 d, and 15 d after inoculation, respectively; and Con_6h, Con_6d, and Con_15d represent leaf samples from the control collected at 6 h, 6 d, and 15 d after inoculation, respectively. "/"represents no DEG enriched significantly in the pairwise comparison.

| No. | gene ID | KO ID | Gene product | KEGG definition | Gene description | $Log_2FC$ | | | | | |
|---|---|---|---|---|---|---|---|---|---|---|---|
| | | | | | | R2-21_ 6h vs. Con_6h | R2-21_ 6d vs. Con_6d | R2-21_15 d vs. Con_15d | XHYN2 _6h vs. Con_6h | XHYN2 _6d vs. Con_6d | XHYN2_ 15d vs. Con_15d |
| 1 | *VIT_16s0039g01240* | K10775 | PAL | phenylalanine ammonia-lyase [EC:4.3.1.24] | Phenylalanine ammonia-lyase | 2.21 | 4.40 | 7.75 | 2.91 | 4.02 | 9.46 |
| 2 | *VIT_16s0039g01110* | K10775 | PAL | phenylalanine ammonia-lyase [EC:4.3.1.24] | Phenylalanine ammonia-lyase | 2.11 | 3.71 | 6.02 | 2.80 | 3.50 | 7.65 |
| 3 | *VIT_16s0039g01280* | K10775 | PAL | phenylalanine ammonia-lyase [EC:4.3.1.24] | Phenylalanine ammonia-lyase | 2.40 | 4.22 | 6.83 | 3.39 | 3.80 | 8.57 |
| 4 | *VIT_16s0039g01300* | K10775 | PAL | phenylalanine ammonia-lyase [EC:4.3.1.24] | Phenylalanine ammonia-lyase | 1.86 | 4.90 | 6.71 | 2.69 | 4.29 | 8.73 |
| 5 | *VIT_00s2849g00010* | K10775 | PAL | phenylalanine ammonia-lyase [EC:4.3.1.24] | Phenylalanine ammonia-lyase | 1.91 | 2.83 | 7.15 | 4.51 | 3.97 | 9.12 |
| 6 | *VIT_16s0039g01360* | K10775 | PAL | phenylalanine ammonia-lyase [EC:4.3.1.24] | Phenylalanine ammonia-lyase | 1.65 | 4.92 | 8.13 | 2.64 | 3.92 | 10.41 |
| 7 | *VIT_16s0039g01120* | K10775 | PAL | phenylalanine ammonia-lyase [EC:4.3.1.24] | Phenylalanine ammonia-lyase | 1.82 | 3.53 | 5.29 | 2.48 | 3.38 | 7.43 |
| 8 | *VIT_16s0039g01170* | K10775 | PAL | phenylalanine ammonia-lyase | Phenylalanine ammonia-lyase | 1.64 | 3.28 | 6.10 | 2.28 | 3.38 | 7.54 |

| | | | | | | | | | | | |
|---|---|---|---|---|---|---|---|---|---|---|---|
| | | | | [EC:4.3.1.24] | | | | | | | |
| 9 | *VIT_00s2508g00010* | K10775 | PAL | phenylalanine ammonia-lyase [EC:4.3.1.24] | Phenylalanine ammonia-lyase | 1.86 | 4.45 | 6.34 | 2.74 | 3.87 | 8.20 |
| 10 | *VIT_16s0039g01100* | K10775 | PAL | phenylalanine ammonia-lyase [EC:4.3.1.24] | Phenylalanine ammonia-lyase | 1.53 | 2.97 | 5.46 | 2.12 | 2.92 | 7.44 |
| 11 | *VIT_16s0039g01130* | K10775 | PAL | phenylalanine ammonia-lyase [EC:4.3.1.24] | Phenylalanine ammonia-lyase | 2.95 | 3.72 | 2.25 | 6.91 | 9.18 | 3.86 |
| 12 | *VIT_08s0040g01710* | K10775 | PAL | phenylalanine ammonia-lyase [EC:4.3.1.24] | Phenylalanine ammonia-lyase | 1.51 | 2.21 | 1.85 | 2.36 | 1.97 | 2.81 |
| 13 | *VIT_13s0019g04460* | K10775 | PAL | phenylalanine ammonia-lyase [EC:4.3.1.24] | Phenylalanine ammonia-lyase | / | 2.13 | 1.09 | 1.72 | 1.87 | 1.70 |
| 14 | *VIT_16s0039g01320* | K10775 | PAL | phenylalanine ammonia-lyase [EC:4.3.1.24] | Phenylalanine ammonia-lyase | / | 3.72 | 6.20 | / | / | 8.79 |
| 15 | *VIT_01s0010g01970* | K00430 | POD | peroxidase [EC:1.11.1.7] | Lignin-forming anionic peroxidase | 4.79 | 4.51 | 3.91 | 5.13 | 5.17 | 4.45 |
| 16 | *VIT_06s0004g07770* | K00430 | POD | peroxidase [EC:1.11.1.7] | Peroxidase 4 | 2.23 | 1.81 | 2.41 | 3.25 | 2.15 | 2.80 |
| 17 | *VIT_18s0001g06850* | K00430 | POD | peroxidase [EC:1.11.1.7] | Cationic peroxidase 1 | / | 2.36 | 2.08 | 1.56 | 2.48 | 1.72 |
| 18 | *VIT_14s0060g00510* | K00430 | POD | peroxidase [EC:1.11.1.7] | Peroxidase 5 | / | / | 4.25 | / | 5.03 | / |
| 19 | *VIT_01s0010g01950* | K00430 | POD | peroxidase [EC:1.11.1.7] | Lignin-forming anionic peroxidase | / | / | / | 4.76 | 5.71 | 3.98 |
| 20 | *VIT_04s0023g02570* | K00430 | POD | peroxidase [EC:1.11.1.7] | Peroxidase 72 | 2.90 | / | / | 2.63 | / | / |
| 21 | *VIT_08s0058g00980* | K00430 | POD | peroxidase [EC:1.11.1.7] | Cationic peroxidase 1 | 2.13 | / | 1.90 | 2.73 | / | 1.41 |
| 22 | *VIT_04s0008g07040* | K00430 | POD | peroxidase [EC:1.11.1.7] | Peroxidase 18 | 5.33 | / | / | 4.35 | / | / |
| 23 | *VIT_01s0010g02020* | K00430 | POD | peroxidase [EC:1.11.1.7] | Lignin-forming anionic peroxidase | 4.82 | 8.42 | / | / | 8.28 | 8.28 |
| 24 | *VIT_01s0010g00390* | K00430 | POD | peroxidase [EC:1.11.1.7] | Peroxidase 7 | / | 5.18 | / | / | / | 4.93 |
| 25 | *VIT_14s0068g01920* | K00430 | POD | peroxidase [EC:1.11.1.7] | Peroxidase 55 | / | 2.20 | 4.14 | / | 2.68 | 5.01 |
| 26 | *VIT_18s0072g00160* | K00430 | POD | peroxidase [EC:1.11.1.7] | Peroxidase 12 | / | 2.91 | / | / | 2.79 | 3.94 |
| 27 | *VIT_08s0058g00990* | K00430 | POD | peroxidase [EC:1.11.1.7] | Cationic peroxidase 1 | / | 3.01 | 5.68 | / | 4.28 | 6.96 |
| 28 | *VIT_12s0055g01020* | K00430 | POD | peroxidase [EC:1.11.1.7] | Peroxidase N1 | 1.57 | 5.20 | / | 1.86 | / | 7.64 |

| | | | | | | | | | | | |
|---|---|---|---|---|---|---|---|---|---|---|---|
| 29 | *VIT_12s0028g01840* | K00430 | POD | peroxidase [EC:1.11.1.7] | Peroxidase 52 | 2.43 | / | / | 2.69 | / | / |
| 30 | *VIT_01s0010g02010* | K00430 | POD | peroxidase [EC:1.11.1.7] | Lignin-forming anionic peroxidase | 1.62 | 9.27 | / | 2.97 | 8.52 | 9.77 |
| 31 | *VIT_07s0129g00360* | K00430 | POD | peroxidase [EC:1.11.1.7] | Peroxidase 73 | / | 1.69 | 2.58 | / | 2.25 | 3.56 |
| 32 | *VIT_12s0055g01030* | K00430 | POD | peroxidase [EC:1.11.1.7] | Peroxidase N1 | / | / | 4.19 | 2.13 | / | 3.59 |
| 33 | *VIT_01s0010g01980* | K00430 | POD | peroxidase [EC:1.11.1.7] | Lignin-forming anionic peroxidase | 5.05 | / | / | / | / | 4.48 |
| 34 | *VIT_12s0055g01000* | K00430 | POD | peroxidase [EC:1.11.1.7] | Peroxidase N1 | 3.78 | / | / | 3.39 | / | / |
| 35 | *VIT_18s0001g06890* | K00430 | POD | peroxidase [EC:1.11.1.7] | Cationic peroxidase 1 | 1.77 | / | / | 2.16 | / | / |
| 36 | *VIT_08s0058g00970* | K00430 | POD | peroxidase [EC:1.11.1.7] | Cationic peroxidase 1 | / | / | / | 3.04 | / | / |
| 37 | *VIT_01s0026g00830* | K00430 | POD | peroxidase [EC:1.11.1.7] | Peroxidase 65 | / | / | 10.32 | / | / | 10.24 |
| 38 | *VIT_05s0020g02120* | K00430 | POD | peroxidase [EC:1.11.1.7] | Peroxidase 24 | / | / | / | / | / | 9.36 |
| 39 | *VIT_06s0004g07740* | K00430 | POD | peroxidase [EC:1.11.1.7] | Cationic peroxidase 1 | / | / | / | / | / | 6.68 |
| 40 | *VIT_12s0055g01010* | K00430 | POD | peroxidase [EC:1.11.1.7] | Peroxidase N1 | / | / | / | 3.33 | / | / |
| 41 | *VIT_12s0055g00990* | K00430 | POD | peroxidase [EC:1.11.1.7] | Peroxidase N1 | / | / | / | 3.40 | / | / |
| 42 | *VIT_01s0010g01960* | K00430 | POD | peroxidase [EC:1.11.1.7] | Lignin-forming anionic peroxidase | / | / | / | / | / | 4.83 |
| 43 | *VIT_12s0055g01070* | K00430 | POD | peroxidase [EC:1.11.1.7] | Cationic peroxidase 2 | / | / | / | / | 5.32 | 7.51 |
| 44 | *VIT_06s0004g01190* | K00430 | POD | peroxidase [EC:1.11.1.7] | Lignin-forming anionic peroxidase | / | / | / | 3.98 | / | / |
| 45 | *VIT_07s0130g00220* | K00430 | POD | peroxidase [EC:1.11.1.7] | Peroxidase 47 | / | / | / | 2.08 | / | / |
| 46 | *VIT_11s0016g05320* | K00430 | POD | peroxidase [EC:1.11.1.7] | Peroxidase 25 | -2.07 | / | / | -1.39 | / | / |
| 47 | *VIT_12s0055g00810* | K00430 | POD | peroxidase [EC:1.11.1.7] | Peroxidase 43 | / | -1.31 | / | / | -1.13 | / |
| 48 | *VIT_12s0059g02420* | K00430 | POD | peroxidase [EC:1.11.1.7] | Peroxidase 3 | / | -2.86 | / | / | -3.60 | -2.79 |
| 49 | *VIT_07s0191g00050* | K00430 | POD | peroxidase [EC:1.11.1.7] | Peroxidase 17 | / | / | / | / | / | -1.27 |
| 50 | *VIT_11s0016g05280* | K00430 | POD | peroxidase [EC:1.11.1.7] | Peroxidase 25 | / | / | / | -2.29 | -2.02 | -4.44 |
| 51 | *VIT_18s0001g06840* | K00430 | POD | peroxidase [EC:1.11.1.7] | Cationic peroxidase 1 | / | / | / | / | / | -1.19 |
| 52 | *VIT_02s0012g00540* | K00430 | POD | peroxidase [EC:1.11.1.7] | Peroxidase 20 | 1.10 | / | -5.42 | / | / | -2.79 |
| 53 | *VIT_06s0004g01440* | K01188 | bglA | beta-glucosidase [EC:3.2.1.21] | Beta-glucosidase 10 | / | / | / | / | / | 8.15 |
| 54 | *VIT_06s0004g01420* | K01188 | bglA | beta-glucosidase [EC:3.2.1.21] | Beta-glucosidase 12 | / | 1.02 | / | / | / | / |
| 55 | *VIT_06s0004g01430* | K01188 | bglA | beta-glucosidase [EC:3.2.1.21] | Beta-glucosidase 12 | / | 2.61 | 4.03 | / | 2.22 | 4.47 |
| 56 | *VIT_07s0005g00360* | K01188 | bglA | beta-glucosidase [EC:3.2.1.21] | Beta-glucosidase 11 | / | 1.04 | / | / | / | / |

| | | | | | | | | | | | |
|---|---|---|---|---|---|---|---|---|---|---|---|
| 57 | *VIT_17s0000g01760* | K01188 | bglA | beta-glucosidase [EC:3.2.1.21] | Beta-glucosidase 24 | / | / | -1.71 | / | / | -2.09 |
| 58 | *VIT_07s0005g00390* | K01188 | bglA | beta-glucosidase [EC:3.2.1.21] | Beta-glucosidase 11 | / | / | / | / | / | -1.36 |
| 59 | *VIT_13s0064g01750* | K01188 | bglA | beta-glucosidase [EC:3.2.1.21] | Beta-glucosidase 13 | / | / | / | / | -2.25 | / |
| 60 | *VIT_13s0064g00520* | K01188 | bglA | beta-glucosidase [EC:3.2.1.21] | Furcatin hydrolase | / | / | -5.60 | / | / | -5.39 |
| 61 | *VIT_01s0011g00760* | K01188 | bglA | beta-glucosidase [EC:3.2.1.21] | Beta-glucosidase 40 | / | / | / | / | / | -2.11 |
| 62 | *VIT_06s0009g00810* | K01188 | bglA | beta-glucosidase [EC:3.2.1.21] | Beta-glucosidase BoGH3B | / | -1.19 | / | / | -1.25 | -1.87 |
| 63 | *VIT_13s0064g01660* | K01188 | bglA | beta-glucosidase [EC:3.2.1.21] | Beta-glucosidase 13 | 3.66 | -3.07 | / | / | -4.48 | / |
| 64 | *VIT_19s0014g03250* | K05350 | bglB | beta-glucosidase [EC:3.2.1.21] | Beta-glucosidase 18 | / | / | / | / | / | -1.09 |
| 65 | *VIT_10s0116g00780* | K05350 | bglB | beta-glucosidase [EC:3.2.1.21] | Beta-glucosidase 46 | / | / | / | / | / | -1.30 |
| 66 | *VIT_17s0000g02680* | K05350 | bglB | beta-glucosidase [EC:3.2.1.21] | Beta-glucosidase 44 | / | -1.17 | / | / | -1.64 | -1.52 |
| 67 | *VIT_08s0032g00470* | K05349 | bglX | beta-glucosidase [EC:3.2.1.21] | Beta-glucosidase BoGH3B | / | 1.94 | 2.05 | / | 2.13 | 2.07 |
| 68 | *VIT_06s0009g00800* | K05349 | bglX | beta-glucosidase [EC:3.2.1.21] | Beta-glucosidase BoGH3B | / | / | 5.44 | / | / | 6.34 |
| 69 | *VIT_00s0615g00020* | K00083 | CAD | cinnamyl-alcohol dehydrogenase [EC:1.1.1.195] | Probable mannitol dehydrogenase | 1.11 | / | 2.93 | / | / | 3.78 |
| 70 | *VIT_10s0003g04910* | K00083 | CAD | cinnamyl-alcohol dehydrogenase [EC:1.1.1.195] | Probable mannitol dehydrogenase | / | 6.34 | 6.92 | / | / | 7.46 |
| 71 | *VIT_00s0346g00110* | K00083 | CAD | cinnamyl-alcohol dehydrogenase [EC:1.1.1.195] | Probable mannitol dehydrogenase | / | / | 11.49 | / | / | 11.86 |
| 72 | *VIT_00s0371g00050* | K00083 | CAD | cinnamyl-alcohol dehydrogenase [EC:1.1.1.195] | Probable mannitol dehydrogenase | 2.58 | 2.99 | 8.26 | / | / | 8.51 |

| | | | | | | | | | | | |
|---|---|---|---|---|---|---|---|---|---|---|---|
| 73 | *VIT_00s1389g00010* | K00083 | CAD | cinnamyl-alcohol dehydrogenase [EC:1.1.1.195] | Probable mannitol dehydrogenase | 2.12 | / | 6.16 | 1.99 | / | 6.59 |
| 74 | *VIT_00s0218g00010* | K00083 | CAD | cinnamyl-alcohol dehydrogenase [EC:1.1.1.195] | Probable mannitol dehydrogenase | / | 1.80 | 2.55 | / | / | 2.97 |
| 75 | *VIT_00s0615g00010* | K00083 | CAD | cinnamyl-alcohol dehydrogenase [EC:1.1.1.195] | Probable mannitol dehydrogenase | 2.65 | / | 4.58 | 3.10 | / | 4.37 |
| 76 | *VIT_18s0122g00450* | K00083 | CAD | cinnamyl-alcohol dehydrogenase [EC:1.1.1.195] | Probable mannitol dehydrogenase | / | 4.61 | / | / | / | / |
| 77 | *VIT_18s0001g14910* | K00083 | CAD | cinnamyl-alcohol dehydrogenase [EC:1.1.1.195] | Probable cinnamyl alcohol dehydrogenase 6 | 3.91 | / | / | 3.92 | / | / |
| 78 | *VIT_00s0615g00030* | K00083 | CAD | cinnamyl-alcohol dehydrogenase [EC:1.1.1.195] | Probable mannitol dehydrogenase | 1.34 | / | / | / | / | / |
| 79 | *VIT_00s0346g00090* | K00083 | CAD | cinnamyl-alcohol dehydrogenase [EC:1.1.1.195] | Probable mannitol dehydrogenase | / | -1.41 | / | / | / | / |
| 80 | *VIT_00s0346g00080* | K00083 | CAD | cinnamyl-alcohol dehydrogenase [EC:1.1.1.195] | Probable mannitol dehydrogenase | / | / | / | / | / | -2.52 |
| 81 | *VIT_04s0044g00190* | K00083 | CAD | cinnamyl-alcohol dehydrogenase [EC:1.1.1.195] | Probable mannitol dehydrogenase | / | / | / | / | / | -1.46 |
| 82 | *VIT_07s0129g01030* | K00083 | CAD | cinnamyl-alcohol dehydrogenase [EC:1.1.1.195] | Probable cinnamyl alcohol dehydrogenase | -1.53 | / | / | / | / | / |
| 83 | *VIT_00s0371g00100* | K00083 | CAD | cinnamyl-alcohol dehydrogenase [EC:1.1.1.195] | Probable mannitol dehydrogenase | -1.70 | / | / | / | / | / |
| 84 | *VIT_11s0052g01090* | K01904 | 4CL | 4-coumarate--CoA ligase [EC:6.2.1.12] | 4-coumarate--CoA ligase 1 | / | 1.64 | / | / | 1.43 | 2.10 |
| 85 | *VIT_06s0061g00450* | K01904 | 4CL | 4-coumarate--CoA ligase | 4-coumarate--CoA | / | 1.38 | 2.39 | / | 1.27 | 2.95 |

| | | | | | | | | | | | |
|---|---|---|---|---|---|---|---|---|---|---|---|
| | | | | [EC:6.2.1.12] | ligase-like 7 | | | | | | |
| 86 | *VIT_11s0052g01110* | K01904 | 4CL | 4-coumarate--CoA ligase [EC:6.2.1.12] | 4-coumarate--CoA ligase 1 | / | 3.76 | 6.47 | / | 2.51 | 7.71 |
| 87 | *VIT_16s0039g02040* | K01904 | 4CL | 4-coumarate--CoA ligase [EC:6.2.1.12] | 4-coumarate--CoA ligase 2 | / | / | / | 1.22 | / | / |
| 88 | *VIT_02s0025g02920* | K13066 | COMT | caffeic acid 3-O-methyltransferase / acetylserotonin O-methyltransferase [EC:2.1.1.68 2.1.1.4] | Caffeic acid 3-O-methyltransferase | 2.03 | 3.53 | 3.66 | 2.17 | 3.12 | 3.84 |
| 89 | *VIT_16s0098g00850* | K13066 | COMT | caffeic acid 3-O-methyltransferase / acetylserotonin O-methyltransferase [EC:2.1.1.68 2.1.1.4] | Caffeic acid 3-O-methyltransferase 1 | 1.33 | 1.01 | / | 1.93 | 1.06 | / |
| 90 | *VIT_15s0048g02460* | K13066 | COMT | caffeic acid 3-O-methyltransferase / acetylserotonin O-methyltransferase [EC:2.1.1.68 2.1.1.4] | Caffeic acid 3-O-methyltransferase 1 | / | / | 3.00 | / | 3.13 | 3.18 |
| 91 | *VIT_19s0135g00030* | K13066 | COMT | caffeic acid 3-O-methyltransferase / acetylserotonin O-methyltransferase [EC:2.1.1.68 2.1.1.4] | Caffeic acid 3-O-methyltransferase | / | 1.72 | / | / | / | 3.50 |
| 92 | *VIT_15s0048g02490* | K13066 | COMT | caffeic acid 3-O-methyltransferase / acetylserotonin O-methyltransferase [EC:2.1.1.68 2.1.1.4] | Flavone 3'-O-methyltransferase 1 | / | 2.04 | 3.51 | / | 3.30 | 3.75 |
| 93 | *VIT_11s0065g00350* | K00487 | CYP73A | trans-cinnamate 4-monooxygenase [EC:1.14.14.91] | Cytochrome P450 CYP73A100 | 2.16 | 4.16 | 5.02 | 2.87 | 4.28 | 7.39 |
| 94 | *VIT_11s0078g00290* | K00487 | CYP73A | trans-cinnamate 4-monooxygenase [EC:1.14.14.91] | Cytochrome P450 CYP73A100 | / | 4.93 | 6.42 | 1.92 | 5.41 | 7.96 |

| | | | | | | | | | | | |
|---|---|---|---|---|---|---|---|---|---|---|---|
| 95 | *VIT_06s0004g08150* | K00487 | CYP73A | trans-cinnamate 4-monooxygenase [EC:1.14.14.91] | Trans-cinnamate 4-monooxygenase | / | 1.23 | / | 1.45 | 1.27 | 1.99 |
| 96 | *VIT_07s0031g00350* | K00588 | CCoAOMT | caffeoyl-CoA O-methyltransferase [EC:2.1.1.104] | Caffeoyl-CoA O-methyltransferase | / | 1.74 | 1.72 | 2.05 | 1.68 | 2.56 |
| 97 | *VIT_03s0063g00140* | K00588 | CCoAOMT | caffeoyl-CoA O-methyltransferase [EC:2.1.1.104] | Caffeoyl-CoA O-methyltransferase | / | / | / | / | / | 1.41 |
| 98 | *VIT_12s0028g03110* | K00588 | CCoAOMT | caffeoyl-CoA O-methyltransferase [EC:2.1.1.104] | Caffeoyl-CoA O-methyltransferase | / | / | / | / | / | -1.09 |
| 99 | *VIT_11s0037g00440* | K13065 | HCT | shikimate O-hydroxycinnamoyltransferase [EC:2.3.1.133] | Shikimate O-hydroxycinnamoyltransferase | / | 1.37 | 2.08 | / | 1.03 | 2.77 |
| 100 | *VIT_09s0018g01190* | K13065 | HCT | shikimate O-hydroxycinnamoyltransferase [EC:2.3.1.133] | Shikimate O-hydroxycinnamoyltransferase | / | / | / | 1.32 | / | / |
| 101 | *VIT_09s0070g00240* | K09753 | CCR | cinnamoyl-CoA reductase [EC:1.2.1.44] | Cinnamoyl-CoA reductase 1 | / | / | / | 1.69 | / | / |
| 102 | *VIT_05s0020g00600* | K11188 | PRDX6 | peroxidase [EC:1.11.1.7] | 1-Cys peroxiredoxin | / | / | 6.75 | / | / | 6.94 |
| 103 | *VIT_04s0023g02900* | K09755 | CYP84A, F5H | ferulate-5-hydroxylase [EC:1.14.-.-] | Cytochrome P450 84A1 | 1.95 | 3.26 | 2.89 | 2.12 | 2.99 | 3.54 |
| 104 | *VIT_08s0040g00780* | K09754 | CYP98A, C3'H | 5-O-(4-coumaroyl)-D-quinate 3'-monooxygenase [EC:1.14.14.96] | Cytochrome P450 98A2 | / | / | / | 1.21 | / | / |
| 105 | *VIT_04s0023g01290* | K12356 | UGT72E | coniferyl-alcohol glucosyltransferase [EC:2.4.1.111] | Anthocyanidin 3-O-glucosyltransferase 5 | 3.75 | / | 4.27 | 4.35 | 3.16 | 2.86 |
| 106 | *VIT_10s0116g01780* | K00430 | PRXR1 | peroxidase [EC:1.11.1.7] | Peroxidase 42 | / | / | / | / | -2.64 | -2.13 |

**Table S4.** DEGs involved in the flavonoid biosynthesis pathway of the tissue cultured seedlings in response to endophytes fungi Epi R2-21 and Alt XHYN2 inoculation (|$Log_2FC$|≥1; *p*-adjust <0.05). DEGs were analyzed in six pairwise comparisons, R2-21_6h vs. Con_6h comparison, R2-21_6d vs. Con_6d, R2-21_15d vs. Con_15d, XHYN2_6h vs. Con_6h, XHYN2_6d vs. Con_6d, XHYN2_15d vs. Con_15d. R2-21_6h, R2-21_6d, and R2-21_15d represent leaf samples from Epi R2-21 treatments collected at 6 h, 6 d, and 15 d after inoculation, respectively; XHYN2_6h, XHYN2_6d, and XHYN2_15d represent leaf samples from Alt XHYN2 treatments collected at 6 h, 6 d, and 15 d after inoculation, respectively; and Con_6h, Con_6d, and Con_15d represent leaf samples from the control collected at 6 h, 6 d, and 15 d after inoculation, respectively. "/"represents no DEG enriched significantly in the pairwise comparison.

| | | | | | | $Log_2FC$ | | | | | |
|---|---|---|---|---|---|---|---|---|---|---|---|
| **No.** | **gene ID** | **KO ID** | **Gene product** | **KEGG definition** | **Gene description** | **R2-21_6h vs. Con_6h** | **R2-21_6d vs. Con_6d** | **R2-21_15d vs. Con_15d** | **XHYN2_6h vs. Con_6h** | **XHYN2_6d vs. Con_6d** | **XHYN2_15d vs. Con_15d** |
| 1 | *VIT_16s0100g00750* | K00660 | CHS | chalcone synthase [EC:2.3.1.74] | Stilbene synthase 6 | 2.06 | 4.77 | 6.49 | 2.97 | 4.32 | 8.50 |
| 2 | *VIT_16s0100g00990* | K00660 | CHS | chalcone synthase [EC:2.3.1.74] | Stilbene synthase 2 | 1.98 | 3.90 | 6.40 | 2.98 | 3.60 | 7.81 |
| 3 | *VIT_16s0100g00920* | K00660 | CHS | chalcone synthase [EC:2.3.1.74] | Stilbene synthase 4 | 1.97 | 2.87 | 4.02 | 3.03 | 3.51 | 3.95 |
| 4 | *VIT_16s0100g00840* | K00660 | CHS | chalcone synthase [EC:2.3.1.74] | Stilbene synthase 4 | 1.61 | 3.37 | 4.97 | 2.64 | 3.66 | 5.24 |
| 5 | *VIT_16s0100g01140* | K00660 | CHS | chalcone synthase [EC:2.3.1.74] | Stilbene synthase 1 | 1.58 | 3.99 | 6.20 | 2.70 | 3.55 | 7.63 |
| 6 | *VIT_10s0042g00920* | K00660 | CHS | chalcone synthase [EC:2.3.1.74] | Stilbene synthase 1 | 1.95 | 2.84 | 4.04 | 3.02 | 3.50 | 3.93 |
| 7 | *VIT_14s0068g00920* | K00660 | CHS | chalcone synthase [EC:2.3.1.74] | Chalcone synthase | 1.74 | 3.44 | 6.27 | 2.83 | 4.11 | 5.36 |
| 8 | *VIT_16s0100g01010* | K00660 | CHS | chalcone synthase [EC:2.3.1.74] | Stilbene synthase 2 | 1.85 | 3.39 | 7.13 | 2.71 | 3.40 | 8.26 |
| 9 | *VIT_16s0100g00880* | K00660 | CHS | chalcone synthase [EC:2.3.1.74] | Stilbene synthase 4 | 1.57 | 3.33 | 7.98 | 2.72 | 3.12 | 9.79 |
| 10 | *VIT_10s0042g00840* | K00660 | CHS | chalcone synthase [EC:2.3.1.74] | Stilbene synthase 3 | 1.99 | 3.51 | 7.05 | 3.10 | 3.80 | 6.25 |
| 11 | *VIT_16s0100g01190* | K00660 | CHS | chalcone synthase [EC:2.3.1.74] | Stilbene synthase 1 | 1.52 | 4.38 | 8.15 | 2.49 | 3.94 | 9.27 |

| | | | | | | | | | | | |
|---|---|---|---|---|---|---|---|---|---|---|---|
| 12 | *VIT_16s0100g00830* | K00660 | CHS | chalcone synthase [EC:2.3.1.74] | Stilbene synthase 5 | 1.51 | 3.77 | 6.50 | 2.54 | 3.53 | 8.44 |
| 13 | *VIT_16s0100g01170* | K00660 | CHS | chalcone synthase [EC:2.3.1.74] | Stilbene synthase 1 | 1.41 | 3.49 | 7.63 | 2.43 | 3.38 | 8.83 |
| 14 | *VIT_16s0100g00850* | K00660 | CHS | chalcone synthase [EC:2.3.1.74] | Stilbene synthase 4 | 1.61 | 3.50 | 4.99 | 2.55 | 3.55 | 5.69 |
| 15 | *VIT_16s0100g01150* | K00660 | CHS | chalcone synthase [EC:2.3.1.74] | Stilbene synthase 6 | 1.68 | 3.99 | 7.55 | 2.56 | 3.50 | 9.21 |
| 16 | *VIT_16s0100g00930* | K00660 | CHS | chalcone synthase [EC:2.3.1.74] | Stilbene synthase 4 | 1.82 | 3.58 | 5.70 | 3.17 | 3.76 | 5.93 |
| 17 | *VIT_16s0100g00910* | K00660 | CHS | chalcone synthase [EC:2.3.1.74] | Stilbene synthase 5 | 1.61 | 3.49 | 5.81 | 2.35 | 3.34 | 7.23 |
| 18 | *VIT_16s0100g00940* | K00660 | CHS | chalcone synthase [EC:2.3.1.74] | Stilbene synthase 4 | 1.68 | 2.76 | 5.37 | 2.74 | 2.99 | 5.80 |
| 19 | *VIT_16s0100g00770* | K00660 | CHS | chalcone synthase [EC:2.3.1.74] | Stilbene synthase 3 | 1.49 | 4.05 | 7.96 | 2.65 | 3.60 | 9.51 |
| 20 | *VIT_16s0100g01000* | K00660 | CHS | chalcone synthase [EC:2.3.1.74] | Stilbene synthase 4 | 1.66 | 3.74 | 8.18 | 2.75 | 3.58 | 8.72 |
| 21 | *VIT_16s0100g01020* | K00660 | CHS | chalcone synthase [EC:2.3.1.74] | Stilbene synthase 4 | 1.33 | 3.71 | 8.51 | 2.19 | 3.87 | 9.18 |
| 22 | *VIT_16s0100g01040* | K00660 | CHS | chalcone synthase [EC:2.3.1.74] | Stilbene synthase 4 | 1.90 | 4.57 | 7.31 | 2.76 | 4.25 | 8.48 |
| 23 | *VIT_10s0042g00880* | K00660 | CHS | chalcone synthase [EC:2.3.1.74] | Stilbene synthase 3 | 1.96 | 8.98 | 7.29 | 3.00 | 9.21 | 6.03 |
| 24 | *VIT_10s0042g00910* | K00660 | CHS | chalcone synthase [EC:2.3.1.74] | Stilbene synthase 3 | 2.00 | / | 8.71 | 3.04 | 4.61 | 7.66 |
| 25 | *VIT_16s0100g00780* | K00660 | CHS | chalcone synthase [EC:2.3.1.74] | Stilbene synthase 5 | / | 4.06 | 5.96 | 1.88 | 3.87 | 7.81 |
| 26 | *VIT_16s0100g01120* | K00660 | CHS | chalcone synthase [EC:2.3.1.74] | Stilbene synthase 6 | / | 3.43 | 8.01 | / | 3.38 | 10.66 |
| 27 | *VIT_16s0100g01100* | K00660 | CHS | chalcone synthase [EC:2.3.1.74] | Stilbene synthase 1 | / | 4.48 | 7.30 | / | 3.70 | 8.11 |
| 28 | *VIT_16s0100g01070* | K00660 | CHS | chalcone synthase [EC:2.3.1.74] | Stilbene synthase 1 | / | 4.69 | 8.36 | 2.15 | 4.04 | 9.07 |
| 29 | *VIT_16s0100g01110* | K00660 | CHS | chalcone synthase [EC:2.3.1.74] | Stilbene synthase 1 | / | 4.08 | 6.12 | 2.53 | 3.65 | 7.57 |

| | | | | | | | | | | | |
|---|---|---|---|---|---|---|---|---|---|---|---|
| 30 | *VIT_10s0042g00930* | K00660 | CHS | chalcone synthase [EC:2.3.1.74] | Stilbene synthase 1 | / | 3.34 | 4.97 | / | 3.97 | 3.91 |
| 31 | *VIT_10s0042g00850* | K00660 | CHS | chalcone synthase [EC:2.3.1.74] | Stilbene synthase 2 | / | / | 5.37 | 3.84 | / | 4.19 |
| 32 | *VIT_14s0068g00930* | K00660 | CHS | chalcone synthase [EC:2.3.1.74] | Chalcone synthase | / | / | -1.89 | / | / | / |
| 33 | *VIT_05s0136g00260* | K00660 | CHS | chalcone synthase [EC:2.3.1.74] | Chalcone synthase 2 | -2.09 | / | -1.51 | / | / | / |
| 34 | *VIT_16s0100g00810* | K13232; K00660 | STS;CHS | stilbene synthase [EC:2.3.1.95];chalcone synthase [EC:2.3.1.74] | Stilbene synthase 4 | 1.57 | 4.02 | 5.84 | 2.61 | 3.85 | 7.30 |
| 35 | *VIT_16s0100g00960* | K13232; K00660 | STS;CHS | stilbene synthase [EC:2.3.1.95];chalcone synthase [EC:2.3.1.74] | Stilbene synthase 4 | 1.58 | 3.71 | 6.60 | 2.62 | 3.52 | 7.77 |
| 36 | *VIT_16s0100g00950* | K13232; K00660 | STS;CHS | stilbene synthase [EC:2.3.1.95];chalcone synthase [EC:2.3.1.74] | Stilbene synthase 2 | 1.91 | 4.07 | 6.29 | 2.85 | 4.59 | 7.33 |
| 37 | *VIT_16s0100g00860* | K13232; K00660 | STS;CHS | stilbene synthase [EC:2.3.1.95];chalcone synthase [EC:2.3.1.74] | Stilbene synthase 4 | 1.53 | 2.88 | 7.01 | 2.57 | 2.88 | 7.56 |
| 38 | *VIT_16s0100g01130* | K13232; K00660 | STS;CHS | stilbene synthase [EC:2.3.1.95];chalcone synthase [EC:2.3.1.74] | Stilbene synthase 1 | 1.42 | 4.53 | 8.04 | 2.18 | 4.01 | 8.59 |
| 39 | *VIT_10s0042g00870* | K13232; K00660 | STS;CHS | stilbene synthase [EC:2.3.1.95];chalcone synthase [EC:2.3.1.74] | Stilbene synthase 2 | 1.91 | 3.61 | 6.37 | 2.92 | 4.07 | 6.18 |
| 40 | *VIT_10s0042g00890* | K13232; K00660 | STS;CHS | stilbene synthase [EC:2.3.1.95];chalcone synthase [EC:2.3.1.74] | Stilbene synthase 1 | / | / | 5.21 | 3.00 | / | 3.54 |
| 41 | *VIT_16s0100g00800* | K13232; K00660 | STS;CHS | stilbene synthase [EC:2.3.1.95];chalcone synthase [EC:2.3.1.74] | Stilbene synthase 4 | / | / | 4.07 | / | 2.35 | 3.55 |
| 42 | *VIT_06s0009g03040* | K13083 | F3'5'H | flavonoid 3',5'-hydroxylase [EC:1.14.14.81] | Flavonoid 3',5'-hydroxylase 2 | 4.29 | 5.66 | 4.77 | 4.67 | 5.34 | 5.10 |
| 43 | *VIT_06s0009g02970* | K13083 | F3'5'H | flavonoid 3',5'-hydroxylase [EC:1.14.14.81] | Flavonoid 3',5'-hydroxylase 2 | / | 1.12 | / | 2.53 | 1.05 | / |

| | | | | | | | | | | | |
|---|---|---|---|---|---|---|---|---|---|---|---|
| 44 | *VIT_06s0009g03110* | K13083 | F3'5'H | flavonoid 3',5'-hydroxylase [EC:1.14.14.81] | Flavonoid 3',5'-hydroxylase 2 | / | 8.10 | / | / | 8.19 | / |
| 45 | *VIT_08s0007g05160* | K13083 | F3'5'H | flavonoid 3',5'-hydroxylase [EC:1.14.14.81] | Flavonoid 3',5'-hydroxylase 2 | / | / | / | / | / | 1.83 |
| 46 | *VIT_06s0009g02830* | K13083 | F3'5'H | flavonoid 3',5'-hydroxylase [EC:1.14.14.81] | Flavonoid 3',5'-hydroxylase | / | -3.68 | / | / | / | / |
| 47 | *VIT_06s0009g03050* | K13083 | F3'5'H | flavonoid 3',5'-hydroxylase [EC:1.14.14.81] | Flavonoid 3',5'-hydroxylase 2 | / | / | -6.93 | / | / | -6.85 |
| 48 | *VIT_06s0009g03010* | K13083 | F3'5'H | flavonoid 3',5'-hydroxylase [EC:1.14.14.81] | Flavonoid 3',5'-hydroxylase 2 | / | -1.20 | -1.73 | / | / | / |
| 49 | *VIT_06s0009g02840* | K13083 | F3'5'H | flavonoid 3',5'-hydroxylase [EC:1.14.14.81] | Flavonoid 3',5'-hydroxylase 2 | / | / | -1.45 | / | / | -1.09 |
| 50 | *VIT_06s0009g02860* | K13083 | F3'5'H | flavonoid 3',5'-hydroxylase [EC:1.14.14.81] | Flavonoid 3',5'-hydroxylase 2 | / | -1.98 | -2.93 | / | -2.15 | / |
| 51 | *VIT_06s0009g02910* | K13083 | F3'5'H | flavonoid 3',5'-hydroxylase [EC:1.14.14.81] | Flavonoid 3',5'-hydroxylase | / | -4.30 | -5.03 | / | -5.10 | / |
| 52 | *VIT_06s0009g02810* | K13083 | F3'5'H | flavonoid 3',5'-hydroxylase [EC:1.14.14.81] | Flavonoid 3',5'-hydroxylase 2 | / | / | -1.15 | 1.60 | -1.52 | / |
| 53 | *VIT_06s0009g02920* | K13083 | F3'5'H | flavonoid 3',5'-hydroxylase [EC:1.14.14.81] | Flavonoid 3',5'-hydroxylase 2 | 1.10 | / | -1.06 | 1.43 | -1.30 | / |
| 54 | *VIT_11s0065g00350* | K00487 | CYP73A | trans-cinnamate 4-monooxygenase [EC:1.14.14.91] | Cytochrome P450 CYP73A100 | 2.16 | 4.16 | 5.02 | 2.87 | 4.28 | 7.39 |
| 55 | *VIT_06s0004g08150* | K00487 | CYP73A | trans-cinnamate 4-monooxygenase [EC:1.14.14.91] | Trans-cinnamate 4-monooxygenase | / | 1.23 | / | 1.45 | 1.27 | 1.99 |
| 56 | *VIT_11s0078g00290* | K00487 | CYP73A | trans-cinnamate 4-monooxygenase [EC:1.14.14.91] | Cytochrome P450 CYP73A100 | / | 4.93 | 6.42 | 1.92 | 5.41 | 7.96 |
| 57 | *VIT_07s0031g00350* | K00588 | CCoAOMT | caffeoyl-CoA O-methyltransferase [EC:2.1.1.104] | Caffeoyl-CoA O-methyltransferase | / | 1.74 | 1.72 | 2.05 | 1.68 | 2.56 |
| 58 | *VIT_03s0063g00140* | K00588 | CCoAOMT | caffeoyl-CoA O-methyltransferase [EC:2.1.1.104] | Caffeoyl-CoA O-methyltransferase | / | / | / | / | / | 1.41 |
| 59 | *VIT_12s0028g03110* | K00588 | CCoAOM | caffeoyl-CoA | Caffeoyl-CoA | / | / | / | / | / | -1.09 |

| | | | | | | | | | | | |
|---|---|---|---|---|---|---|---|---|---|---|---|
| | | | T | O-methyltransferase [EC:2.1.1.104] | O-methyltransferase | | | | | | |
| 60 | *VIT_18s0001g14310* | K00475 | F3H | naringenin 3-dioxygenase [EC:1.14.11.9] | Flavanone 3-dioxygenase | / | / | 1.75 | / | / | 2.82 |
| 61 | *VIT_04s0023g03370* | K00475 | F3H | naringenin 3-dioxygenase [EC:1.14.11.9] | Naringenin,2-oxoglutarate 3-dioxygenase | -1.50 | / | / | / | / | / |
| 62 | *VIT_18s0001g03510* | K05278 | FLS | flavonol synthase [EC:1.14.20.6] | Flavonol synthase/flavanone 3-hydroxylase | / | 3.86 | 4.44 | / | / | 4.61 |
| 63 | *VIT_18s0001g03430* | K05278 | FLS | flavonol synthase [EC:1.14.20.6] | Flavonol synthase/flavanone 3-hydroxylase | / | 2.51 | / | / | 2.15 | / |
| 64 | *VIT_11s0037g00440* | K13065 | HCT | shikimate O-hydroxycinnamoyltransferase [EC:2.3.1.133] | Shikimate O-hydroxycinnamoyltransferase | / | 1.37 | 2.08 | / | 1.03 | 2.77 |
| 65 | *VIT_09s0018g01190* | K13065 | HCT | shikimate O-hydroxycinnamoyltransferase [EC:2.3.1.133] | Shikimate O-hydroxycinnamoyltransferase | / | / | / | 1.32 | / | / |
| 66 | *VIT_18s0001g12800* | K13082 | DFR | bifunctional dihydroflavonol 4-reductase/flavanone 4-reductase [EC:1.1.1.219 1.1.1.234] | Dihydroflavonol 4-reductase | -1.57 | / | / | / | / | / |
| 67 | *VIT_18s0001g12810* | K13082 | DFR | bifunctional dihydroflavonol 4-reductase/flavanone 4-reductase [EC:1.1.1.219 1.1.1.234] | Dihydroflavonol 4-reductase | / | -1.30 | / | / | / | -1.37 |
| 68 | *VIT_13s0067g03820* | K01859 | CHI | chalcone isomerase [EC:5.5.1.6] | Chalcone-flavonone isomerase 2 | / | 1.46 | 1.56 | 1.53 | 1.84 | 2.00 |
| 69 | *VIT_02s0025g04720* | K05277 | ANS | anthocyanidin synthase [EC:1.14.20.4] | Leucoanthocyanidin dioxygenase | / | 1.02 | / | / | 1.08 | 1.71 |
| 70 | *VIT_08s0040g00780* | K09754 | CYP98A, C3'H | 5-O-(4-coumaroyl)-D-quinate 3'-monooxygenase [EC:1.14.14.96] | Cytochrome P450 98A2 | / | / | / | 1.21 | / | / |

**Table S5.** DEGs involved in the phenylalanine metabolism pathway of the tissue cultured seedlings in response to endophytes fungi Epi R2-21 and Alt XHYN2 inoculation (|$Log_2$FC|≥1; *p*-adjust <0.05). DEGs were analyzed in six pairwise comparisons, R2-21_6h vs. Con_6h comparison, R2-21_6d vs. Con_6d, R2-21_15d vs. Con_15d, XHYN2_6h vs. Con_6h, XHYN2_6d vs. Con_6d, XHYN2_15d vs. Con_15d. R2-21_6h, R2-21_6d, and R2-21_15d represent leaf samples from Epi R2-21 treatments collected at 6 h, 6 d, and 15 d after inoculation, respectively; XHYN2_6h, XHYN2_6d, and XHYN2_15d represent leaf samples from Alt XHYN2 treatments collected at 6 h, 6 d, and 15 d after inoculation, respectively; and Con_6h, Con_6d, and Con_15d represent leaf samples from the control collected at 6 h, 6 d, and 15 d after inoculation, respectively. "/"represents no DEG enriched significantly in the pairwise comparison.

| **No.** | **gene ID** | **KO ID** | **Gene product** | **KEGG definition** | **Gene description** | **$Log_2$FC** | | | | | |
|---|---|---|---|---|---|---|---|---|---|---|---|
| | | | | | | **R2-21_ 6h vs. Con_6h** | **R2-21_ 6d vs. Con_6d** | **R2-21_15 d vs. Con_15d** | **XHYN2 _6h vs. Con_6h** | **XHYN2 _6d vs. Con_6d** | **XHYN2_ 15d vs. Con_15d** |
| 1 | *VIT_16s0039g01130* | K10775 | PAL | phenylalanine ammonia-lyase [EC:4.3.1.24] | Phenylalanine ammonia-lyase | 2.25 | 3.86 | 6.91 | 2.95 | 3.72 | 9.18 |
| 2 | *VIT_16s0039g01280* | K10775 | PAL | phenylalanine ammonia-lyase [EC:4.3.1.24] | Phenylalanine ammonia-lyase | 2.40 | 4.22 | 6.83 | 3.39 | 3.80 | 8.57 |
| 3 | *VIT_16s0039g01240* | K10775 | PAL | phenylalanine ammonia-lyase [EC:4.3.1.24] | Phenylalanine ammonia-lyase | 2.21 | 4.40 | 7.75 | 2.91 | 4.02 | 9.46 |
| 4 | *VIT_16s0039g01110* | K10775 | PAL | phenylalanine ammonia-lyase [EC:4.3.1.24] | Phenylalanine ammonia-lyase | 2.11 | 3.71 | 6.02 | 2.80 | 3.50 | 7.65 |
| 5 | *VIT_16s0039g01360* | K10775 | PAL | phenylalanine ammonia-lyase [EC:4.3.1.24] | Phenylalanine ammonia-lyase | 1.65 | 4.92 | 8.13 | 2.64 | 3.92 | 10.41 |
| 6 | *VIT_16s0039g01170* | K10775 | PAL | phenylalanine ammonia-lyase [EC:4.3.1.24] | Phenylalanine ammonia-lyase | 1.64 | 3.28 | 6.10 | 2.28 | 3.38 | 7.54 |
| 7 | *VIT_16s0039g01300* | K10775 | PAL | phenylalanine ammonia-lyase [EC:4.3.1.24] | Phenylalanine ammonia-lyase | 1.86 | 4.90 | 6.71 | 2.69 | 4.29 | 8.73 |
| 8 | *VIT_00s2849g00010* | K10775 | PAL | phenylalanine ammonia-lyase [EC:4.3.1.24] | Phenylalanine ammonia-lyase | 1.91 | 2.83 | 7.15 | 4.51 | 3.97 | 9.12 |
| 9 | *VIT_00s2508g00010* | K10775 | PAL | phenylalanine ammonia-lyase [EC:4.3.1.24] | Phenylalanine ammonia-lyase | 1.86 | 4.45 | 6.34 | 2.74 | 3.87 | 8.20 |
| 10 | *VIT_16s0039g01100* | K10775 | PAL | phenylalanine ammonia-lyase [EC:4.3.1.24] | Phenylalanine ammonia-lyase | 1.53 | 2.97 | 5.46 | 2.12 | 2.92 | 7.44 |
| 11 | *VIT_16s0039g01120* | K10775 | PAL | phenylalanine ammonia-lyase [EC:4.3.1.24] | Phenylalanine ammonia-lyase | 1.82 | 3.53 | 5.29 | 2.48 | 3.38 | 7.43 |

| | | | | | | | | | | | |
|---|---|---|---|---|---|---|---|---|---|---|---|
| 12 | *VIT_08s0040g01710* | K10775 | PAL | phenylalanine ammonia-lyase [EC:4.3.1.24] | Phenylalanine ammonia-lyase | 1.51 | 2.21 | 1.85 | 2.36 | 1.97 | 2.81 |
| 13 | *VIT_13s0019g04460* | K10775 | PAL | phenylalanine ammonia-lyase [EC:4.3.1.24] | Phenylalanine ammonia-lyase | / | 2.13 | 1.09 | 1.72 | 1.87 | 1.70 |
| 14 | *VIT_06s0061g00450* | K01904 | 4CL | 4-coumarate--CoA ligase [EC:6.2.1.12] | 4-coumarate--CoA ligase-like 7 | / | 1.38 | 2.39 | / | 1.27 | 2.95 |
| 15 | *VIT_11s0052g01110* | K01904 | 4CL | 4-coumarate--CoA ligase [EC:6.2.1.12] | 4-coumarate--CoA ligase 1 | / | 3.76 | 6.47 | / | 2.51 | 7.71 |
| 16 | *VIT_11s0052g01090* | K01904 | 4CL | 4-coumarate--CoA ligase [EC:6.2.1.12] | 4-coumarate--CoA ligase 1 | / | 1.64 | / | / | 1.43 | 2.10 |
| 17 | *VIT_16s0039g01320* | K10775 | PAL | phenylalanine ammonia-lyase [EC:4.3.1.24] | Phenylalanine ammonia-lyase | / | 3.72 | 6.20 | / | / | 8.79 |
| 18 | *VIT_16s0039g02040* | K01904 | 4CL | 4-coumarate--CoA ligase [EC:6.2.1.12] | 4-coumarate--CoA ligase 2 | / | / | / | 1.22 | / | / |
| 19 | *VIT_05s0020g03280* | K00276 | AOC3 | primary-amine oxidase [EC:1.4.3.21] | Primary amine oxidase | / | 3.07 | 4.46 | / | / | 5.84 |
| 20 | *VIT_00s0225g00090* | K00276 | AOC3 | primary-amine oxidase [EC:1.4.3.21] | Primary amine oxidase | 1.86 | / | 2.17 | 1.67 | / | 2.96 |
| 21 | *VIT_17s0000g09100* | K00276 | AOC3 | primary-amine oxidase [EC:1.4.3.21] | Primary amine oxidase | / | / | / | / | / | 1.58 |
| 22 | *VIT_05s0094g01210* | K00276 | AOC3 | primary-amine oxidase [EC:1.4.3.21] | Primary amine oxidase | 1.64 | / | / | / | / | / |
| 23 | *VIT_00s1937g00010* | K00276 | AOC3 | primary-amine oxidase [EC:1.4.3.21] | Primary amine oxidase | 1.35 | / | / | / | / | / |
| 24 | *VIT_05s0020g03260* | K00276 | AOC3 | primary-amine oxidase [EC:1.4.3.21] | Primary amine oxidase | / | / | 5.69 | / | / | / |
| 25 | *VIT_00s1682g00010* | K00276 | AOC3 | primary-amine oxidase [EC:1.4.3.21] | Primary amine oxidase | 1.63 | / | / | 1.21 | / | / |
| 26 | *VIT_05s0020g03310* | K00276 | AOC3 | primary-amine oxidase [EC:1.4.3.21] | Primary amine oxidase 1 | / | / | -2.03 | / | / | -1.43 |
| 27 | *VIT_11s0065g00350* | K00487 | CYP73A | trans-cinnamate 4-monooxygenase [EC:1.14.14.91] | Cytochrome P450 CYP73A100 | 2.16 | 4.16 | 5.02 | 2.87 | 4.28 | 7.39 |
| 28 | *VIT_11s0078g00290* | K00487 | CYP73A | trans-cinnamate 4-monooxygenase [EC:1.14.14.91] | Cytochrome P450 CYP73A100 | / | 4.93 | 6.42 | 1.92 | 5.41 | 7.96 |

| | | | | | | | | | | | |
|---|---|---|---|---|---|---|---|---|---|---|---|
| 29 | *VIT_06s0004g08150* | K00487 | CYP73A | trans-cinnamate 4-monooxygenase [EC:1.14.14.91] | Trans-cinnamate 4-monooxygenase | / | 1.23 | / | 1.45 | 1.27 | 1.99 |
| 30 | *VIT_19s0014g02190* | K00815 | TAT | tyrosine aminotransferase [EC:2.6.1.5] | Probable aminotransferase TAT2 | / | 2.73 | 3.14 | / | 2.43 | 3.94 |
| 31 | *VIT_12s0028g03260* | K00815 | TAT | tyrosine aminotransferase [EC:2.6.1.5] | S-alkyl-thiohydroximate lyase SUR1 | / | 3.23 | / | / | / | / |
| 32 | *VIT_12s0028g03210* | K00815 | TAT | tyrosine aminotransferase [EC:2.6.1.5] | Tyrosine aminotransferase OS | -1.06 | / | / | / | / | / |
| 33 | *VIT_07s0031g00350* | K00588 | CCoAOMT | caffeoyl-CoA O-methyltransferase [EC:2.1.1.104] | Caffeoyl-CoA O-methyltransferase | / | 1.74 | 1.72 | 2.05 | 1.68 | 2.56 |
| 34 | *VIT_12s0028g03110* | K00588 | CCoAOMT | caffeoyl-CoA O-methyltransferase [EC:2.1.1.104] | Caffeoyl-CoA O-methyltransferase | / | / | / | / | / | -1.09 |
| 35 | *VIT_12s0028g01820* | K14455 | GOT2 | aspartate aminotransferase, mitochondrial [EC:2.6.1.1] | Aspartate aminotransferase, mitochondrial | / | 1.33 | 1.37 | / | 1.05 | 1.94 |
| 36 | *VIT_08s0058g01000* | K14455 | GOT2 | aspartate aminotransferase, mitochondrial [EC:2.6.1.1] | Aspartate aminotransferase, mitochondrial | / | / | / | / | 1.58 | / |
| 37 | *VIT_04s0008g03770* | K14454 | GOT1 | aspartate aminotransferase, cytoplasmic [EC:2.6.1.1] | Aspartate aminotransferase 3, chloroplastic | / | 1.95 | 3.58 | / | 1.77 | 4.17 |
| 38 | *VIT_12s0028g00710* | K00457 | HPD, hppD | 4-hydroxyphenylpyruvate dioxygenase [EC:1.13.11.27] | 4-hydroxyphenylpyruvate dioxygenase | / | 1.78 | 3.56 | / | 1.94 | 4.47 |
| 39 | *VIT_03s0063g00140* | K00588 | ATAMI1 | caffeoyl-CoA O-methyltransferase [EC:2.1.1.104] | Caffeoyl-CoA O-methyltransferase | / | / | / | / | / | 1.41 |
| 40 | *VIT_11s0052g00040* | K07253 | MIF | phenylpyruvate tautomerase [EC:5.3.2.1] | Macrophage migration inhibitory factor homolog | / | / | / | / | / | -1.33 |
| 41 | *VIT_18s0001g04860* | K15849 | PAT, AAT | bifunctional aspartate aminotransferase and glutamate/aspartate-prephenate aminotransferase [EC:2.6.1.1 2.6.1.78 2.6.1.79] | Bifunctional aspartate aminotransferase and glutamate/aspartate-prephenate aminotransferase | / | 1.82 | 1.56 | 1.25 | / | 2.69 |

**Table S6.** DEGs involved in the stilbenoid, diarylheptanoid and gingerol biosynthesis pathway of the tissue cultured seedlings in response to endophytes fungi Epi R2-21 and Alt XHYN2 inoculation (|$Log_2FC$|≥1; *p*-adjust <0.05). DEGs were analyzed in six pairwise comparisons, R2-21_6h vs. Con_6h comparison, R2-21_6d vs. Con_6d, R2-21_15d vs. Con_15d, XHYN2_6h vs. Con_6h, XHYN2_6d vs. Con_6d, XHYN2_15d vs. Con_15d. R2-21_6h, R2-21_6d, and R2-21_15d represent leaf samples from Epi R2-21 treatments collected at 6 h, 6 d, and 15 d after inoculation, respectively; XHYN2_6h, XHYN2_6d, and XHYN2_15d represent leaf samples from Alt XHYN2 treatments collected at 6 h, 6 d, and 15 d after inoculation, respectively; and Con_6h, Con_6d, and Con_15d represent leaf samples from the control collected at 6 h, 6 d, and 15 d after inoculation, respectively. "/"represents no DEG enriched significantly in the pairwise comparison.

| No. | gene ID | KO ID | Gene product | KEGG definition | Gene description | $Log_2FC$ | | | | | |
|---|---|---|---|---|---|---|---|---|---|---|---|
| | | | | | | R2-21_6h vs. Con_6h | R2-21_6d vs. Con_6d | R2-21_15d vs. Con_15d | XHYN2_6h vs. Con_6h | XHYN2_6d vs. Con_6d | XHYN2_15d vs. Con_15d |
| 1 | *VIT_11s0065g00350* | K00487 | CYP73A | trans-cinnamate 4-monooxygenase [EC:1.14.14.91] | Cytochrome P450 CYP73A100 | 2.16 | 4.16 | 5.02 | 2.87 | 4.28 | 7.39 |
| 2 | *VIT_11s0078g00290* | K00487 | CYP73A | trans-cinnamate 4-monooxygenase [EC:1.14.14.91] | Cytochrome P450 CYP73A100 | / | 4.93 | 6.42 | 1.92 | 5.41 | 7.96 |
| 3 | *VIT_06s0004g08150* | K00487 | CYP73A | trans-cinnamate 4-monooxygenase [EC:1.14.14.91] | Trans-cinnamate 4-monooxygenase | / | 1.23 | / | 1.45 | 1.27 | 1.99 |
| 4 | *VIT_16s0100g00810* | K13232; K00660 | STS;CHS | stilbene synthase [EC:2.3.1.95];chalcone synthase [EC:2.3.1.74] | Stilbene synthase 4 | 1.57 | 4.02 | 5.84 | 2.61 | 3.85 | 7.30 |
| 5 | *VIT_16s0100g00960* | K13232; K00660 | STS;CHS | stilbene synthase [EC:2.3.1.95];chalcone synthase [EC:2.3.1.74] | Stilbene synthase 4 | 1.58 | 3.71 | 6.60 | 2.62 | 3.52 | 7.77 |
| 6 | *VIT_16s0100g00860* | K13232; K00660 | STS;CHS | stilbene synthase [EC:2.3.1.95];chalcone synthase [EC:2.3.1.74] | Stilbene synthase 4 | 1.53 | 2.88 | 7.01 | 2.57 | 2.88 | 7.56 |
| 7 | *VIT_16s0100g01130* | K13232; K00660 | STS;CHS | stilbene synthase [EC:2.3.1.95];chalcone synthase [EC:2.3.1.74] | Stilbene synthase 1 | 1.42 | 4.53 | 8.04 | 2.18 | 4.01 | 8.59 |
| 8 | *VIT_10s0042g00870* | K13232; | STS;CHS | stilbene synthase | Stilbene synthase 2 | 1.91 | 3.61 | 6.37 | 2.92 | 4.07 | 6.18 |

| | | | | | | | | | | | |
|---|---|---|---|---|---|---|---|---|---|---|---|
| | | K00660 | | [EC:2.3.1.95];chalcone synthase [EC:2.3.1.74] | | | | | | | |
| 9 | *VIT_16s0100g00950* | K13232; K00660 | STS;CHS | stilbene synthase [EC:2.3.1.95];chalcone synthase [EC:2.3.1.74] | Stilbene synthase 2 | 1.91 | 4.07 | 6.29 | 2.85 | 4.59 | 7.33 |
| 10 | *VIT_16s0100g00800* | K13232; K00660 | STS;CHS | stilbene synthase [EC:2.3.1.95];chalcone synthase [EC:2.3.1.74] | Stilbene synthase 4 | / | / | 4.07 | / | 2.35 | 3.55 |
| 11 | *VIT_10s0042g00890* | K13232; K00660 | STS;CHS | stilbene synthase [EC:2.3.1.95];chalcone synthase [EC:2.3.1.74] | Stilbene synthase 1 | / | / | 5.21 | 3.00 | / | 3.54 |
| 12 | *VIT_16s0100g00900* | K13232 | STS | stilbene synthase [EC:2.3.1.95] | Stilbene synthase 4 | 1.41 | 3.57 | 7.68 | 2.54 | 3.35 | 9.48 |
| 13 | *VIT_16s0100g01030* | K13232 | STS | stilbene synthase [EC:2.3.1.95] | Stilbene synthase 3 | 1.80 | 3.97 | 7.60 | 2.65 | 3.32 | 8.82 |
| 14 | *VIT_16s0100g01200* | K13232 | STS | stilbene synthase [EC:2.3.1.95] | Stilbene synthase 6 | 1.46 | 4.54 | 8.38 | 2.14 | 4.10 | 9.03 |
| 15 | *VIT_16s0100g01160* | K13232 | STS | stilbene synthase [EC:2.3.1.95] | Stilbene synthase 1 | / | 3.78 | 5.70 | 2.51 | 3.58 | 7.41 |
| 16 | *VIT_10s0003g00480* | K16040 | ROMT | trans-resveratrol di-O-methyltransferase [EC:2.1.1.240] | Trans-resveratrol di-O-methyltransferase | 2.12 | 3.10 | 3.39 | 2.05 | 3.06 | 3.85 |
| 17 | *VIT_12s0028g01940* | K16040 | ROMT | trans-resveratrol di-O-methyltransferase [EC:2.1.1.240] | Trans-resveratrol di-O-methyltransferase | / | / | 2.56 | / | / | 2.08 |
| 18 | *VIT_12s0028g02890* | K16040 | ROMT | trans-resveratrol di-O-methyltransferase [EC:2.1.1.240] | Trans-resveratrol di-O-methyltransferase | / | / | / | / | / | 1.20 |
| 19 | *VIT_10s0003g00460* | K16040 | ROMT | trans-resveratrol di-O-methyltransferase [EC:2.1.1.240] | Trans-resveratrol di-O-methyltransferase | / | / | / | / | / | 3.75 |
| 20 | *VIT_12s0028g01880* | K16040 | ROMT | trans-resveratrol di-O-methyltransferase [EC:2.1.1.240] | Trans-resveratrol di-O-methyltransferase | / | / | 2.45 | 3.19 | 1.04 | / |
| 21 | *VIT_12s0028g02840* | K16040 | ROMT | trans-resveratrol di-O-methyltransferase | Trans-resveratrol di-O-methyltransferase | / | / | / | / | -2.85 | / |

| | | | | | | | | | | | |
|---|---|---|---|---|---|---|---|---|---|---|---|
| | | | | [EC:2.1.1.240] | | | | | | | |
| 22 | *VIT_10s0003g00440* | K16040; K13262 | ROMT;7-IOMT | trans-resveratrol di-O-methyltransferase [EC:2.1.1.240];isoflavone-7-O-methyltransferase [EC:2.1.1.150] | Trans-resveratrol di-O-methyltransferase | / | / | / | / | / | 3.33 |
| 23 | *VIT_07s0031g00350* | K00588 | CCoAOMT | caffeoyl-CoA O-methyltransferase [EC:2.1.1.104] | Caffeoyl-CoA O-methyltransferase | / | 1.74 | 1.72 | 2.05 | 1.68 | 2.56 |
| 24 | *VIT_03s0063g00140* | K00588 | CCoAOMT | caffeoyl-CoA O-methyltransferase [EC:2.1.1.104] | Caffeoyl-CoA O-methyltransferase | / | / | / | / | / | 1.41 |
| 25 | *VIT_12s0028g03110* | K00588 | CCoAOMT | caffeoyl-CoA O-methyltransferase [EC:2.1.1.104] | Caffeoyl-CoA O-methyltransferase | / | / | / | / | / | -1.09 |
| 26 | *VIT_11s0037g00440* | K13065 | HCT | shikimate O-hydroxycinnamoyltransferase [EC:2.3.1.133] | Shikimate O-hydroxycinnamoyltransferase | / | 1.37 | 2.08 | / | 1.03 | 2.77 |
| 27 | *VIT_09s0018g01190* | K13065 | HCT | shikimate O-hydroxycinnamoyltransferase [EC:2.3.1.133] | Shikimate O-hydroxycinnamoyltransferase | / | / | / | 1.32 | / | / |
| 28 | *VIT_08s0040g00780* | K09754 | CYP98A, C3'H | 5-O-(4-coumaroyl)-D-quinate 3'-monooxygenase [EC:1.14.14.96] | Cytochrome P450 98A2 | / | / | / | 1.21 | / | / |

**Table S7.** DEGs involved in the plant-pathogen interaction pathway of the tissue cultured seedlings in response to endophytes fungi Epi R2-21 and Alt XHYN2 inoculation ($|Log_2FC|\geq1$; *p*-adjust <0.05). DEGs were analyzed in six pairwise comparisons, R2-21_6h vs. Con_6h comparison, R2-21_6d vs. Con_6d, R2-21_15d vs. Con_15d, XHYN2_6h vs. Con_6h, XHYN2_6d vs. Con_6d, XHYN2_15d vs. Con_15d. R2-21_6h, R2-21_6d, and R2-21_15d represent leaf samples from Epi R2-21 treatments collected at 6 h, 6 d, and 15 d after inoculation, respectively; XHYN2_6h, XHYN2_6d, and XHYN2_15d represent leaf samples from Alt XHYN2 treatments collected at 6 h, 6 d, and 15 d after inoculation, respectively; and Con_6h, Con_6d, and Con_15d represent leaf samples from the control collected at 6 h, 6 d, and 15 d after inoculation, respectively. "/"represents no DEG enriched significantly in the pairwise comparison.

| | | | | | | $Log_2FC$ | | | | | |
|---|---|---|---|---|---|---|---|---|---|---|---|
| **No.** | **gene ID** | **KO ID** | **Gene product** | **KEGG definition** | **Gene description** | **R2-21_6h vs. Con_6h** | **R2-21_6d vs. Con_6d** | **R2-21_15d vs. Con_15d** | **XHYN2_6h vs. Con_6h** | **XHYN2_6d vs. Con_6d** | **XHYN2_15d vs. Con_15d** |
| 1 | *VIT_04s0023g01100* | K13448 | CML | calcium-binding protein CML | Probable calcium-binding protein CML41 | 2.07 | 2.44 | 2.86 | 1.85 | 2.75 | 2.84 |
| 2 | *VIT_17s0000g01630* | K13448 | CML | calcium-binding protein CML | Putative calcium-binding protein CML19 | 4.82 | 2.78 | 3.53 | 5.01 | 4.38 | 2.43 |
| 3 | *VIT_01s0010g03010* | K13448 | CML | calcium-binding protein CML | Probable calcium-binding protein CML31 | 3.92 | 2.25 | 6.09 | 5.35 | 4.46 | 5.41 |
| 4 | *VIT_18s0122g00180* | K13448 | CML | calcium-binding protein CML | Calcium-binding protein CML37 | 2.56 | 1.68 | 1.13 | 1.87 | 2.10 | 1.08 |
| 5 | *VIT_00s0179g00280* | K13448 | CML | calcium-binding protein CML | Calmodulin-like protein 8 | 3.59 | 3.61 | 6.53 | 6.17 | 2.36 | 7.36 |
| 6 | *VIT_01s0010g02980* | K13448 | CML | calcium-binding protein CML | Probable calcium-binding protein CML31 | 2.90 | 2.76 | 4.81 | 5.07 | 4.08 | 4.62 |
| 7 | *VIT_01s0010g02970* | K13448 | CML | calcium-binding protein CML | Probable calcium-binding protein CML31 | 2.48 | 2.74 | 5.22 | 4.64 | 3.57 | 4.88 |
| 8 | *VIT_01s0010g02930* | K13448 | CML | calcium-binding protein CML | Probable calcium-binding protein CML31 | 1.65 | 2.05 | 4.38 | 3.91 | 3.41 | 3.48 |
| 9 | *VIT_01s0010g03040* | K13448 | CML | calcium-binding protein CML | Putative calcium-binding protein CML23 | 3.83 | 2.09 | 5.33 | 5.32 | 3.68 | 5.02 |
| 10 | *VIT_08s0056g00290* | K13448 | CML | calcium-binding protein CML | Calcium-binding allergen Bet v3 | 3.78 | 1.79 | 2.10 | 3.90 | 2.16 | 1.87 |
| 11 | *VIT_14s0030g02150* | K13448 | CML | calcium-binding protein CML | Calmodulin-like protein | 1.74 | 1.93 | 3.54 | 2.28 | 1.68 | 4.03 |

| | | | | | | | | | | | |
|---|---|---|---|---|---|---|---|---|---|---|---|
| | | | | | 11 | | | | | | |
| 12 | *VIT_01s0010g03020* | K13448 | CML | calcium-binding protein CML | Probable calcium-binding protein CML31 | 3.33 | 2.38 | 4.66 | 5.57 | 4.18 | 4.26 |
| 13 | *VIT_14s0006g01400* | K13448 | CML | calcium-binding protein CML | Probable calcium-binding protein CML23 | 1.13 | 1.19 | 1.76 | / | 1.42 | 1.25 |
| 14 | *VIT_01s0010g02960* | K13448 | CML | calcium-binding protein CML | Probable calcium-binding protein CML31 | / | 2.50 | 4.48 | 4.00 | 3.55 | 3.73 |
| 15 | *VIT_01s0010g02940* | K13448 | CML | calcium-binding protein CML | Probable calcium-binding protein CML31 | / | 2.57 | 4.11 | 3.74 | 3.48 | 3.38 |
| 16 | *VIT_05s0020g04420* | K13448 | CML | calcium-binding protein CML | Calmodulin-like protein 11 | 1.21 | / | 2.43 | 3.19 | 1.64 | 1.96 |
| 17 | *VIT_06s0080g00450* | K13448 | CML | calcium-binding protein CML | Probable calcium-binding protein CML48 | / | 1.35 | 2.71 | / | 1.66 | 2.27 |
| 18 | *VIT_01s0010g02950* | K13448 | CML | calcium-binding protein CML | Probable calcium-binding protein CML31 | / | 1.60 | 4.82 | 4.00 | 3.02 | 4.16 |
| 19 | *VIT_07s0031g00700* | K13448 | CML | calcium-binding protein CML | Caltractin | / | 1.01 | 1.88 | / | 1.19 | 2.09 |
| 20 | *VIT_16s0039g01880* | K13448 | CML | calcium-binding protein CML | Probable calcium-binding protein CML18 | / | / | / | / | 1.05 | 1.14 |
| 21 | *VIT_02s0012g02060* | K13448 | CML | calcium-binding protein CML | Probable calcium-binding protein CML18 | / | / | / | / | 3.01 | 2.15 |
| 22 | *VIT_01s0010g03000* | K13448 | CML | calcium-binding protein CML | Probable calcium-binding protein CML31 | / | / | / | / | 4.49 | 5.61 |
| 23 | *VIT_14s0171g00150* | K13448 | CML | calcium-binding protein CML | Probable calcium-binding protein CML31 | / | / | / | 3.96 | / | 5.04 |
| 24 | *VIT_01s0011g02470* | K13448 | CML | calcium-binding protein CML | Probable calcium-binding protein CML16 | 1.46 | / | / | 2.06 | 1.26 | / |
| 25 | *VIT_18s0001g11830* | K13448 | CML | calcium-binding protein CML | Probable calcium-binding protein CML41 | / | / | 1.30 | / | / | / |
| 26 | *VIT_05s0102g00450* | K13448 | CML | calcium-binding protein CML | Calmodulin-like protein 5 | / | / | 1.12 | / | / | / |
| 27 | *VIT_01s0010g02990* | K13448 | CML | calcium-binding protein CML | Probable calcium-binding protein CML31 | / | / | 5.21 | / | / | / |
| 28 | *VIT_05s0029g00070* | K13448 | CML | calcium-binding protein CML | Probable calcium-binding protein CML22 | / | / | -1.38 | / | / | -1.13 |
| 29 | *VIT_01s0011g00990* | K13457 | RPM1, RPS3 | disease resistance protein RPM1 | Disease resistance protein RPM1 | 2.49 | 1.40 | 4.04 | 4.16 | 2.66 | 3.48 |
| 30 | *VIT_15s0045g00810* | K13457 | RPM1, | disease resistance protein | Putative disease resistance | / | 1.40 | 2.52 | 2.87 | 1.64 | 1.60 |

| | | | | | | | | | | | |
|---|---|---|---|---|---|---|---|---|---|---|---|
| | | | RPS3 | RPM1 | protein At1g50180 | | | | | | |
| 31 | *VIT_15s0045g00880* | K13457 | RPM1, RPS3 | disease resistance protein RPM1 | Putative disease resistance protein At1g50180 | / | 1.05 | 3.28 | 2.32 | 1.54 | 2.55 |
| 32 | *VIT_15s0046g03660* | K13457 | RPM1, RPS3 | disease resistance protein RPM1 | Disease resistance protein RPH8A | / | 1.63 | 3.05 | 2.56 | 2.61 | 2.22 |
| 33 | *VIT_07s0005g06210* | K13457 | RPM1, RPS3 | disease resistance protein RPM1 | Disease resistance protein RPM1 | / | 1.38 | 2.23 | 1.99 | 1.28 | 1.64 |
| 34 | *VIT_03s0038g01400* | K13457 | RPM1, RPS3 | disease resistance protein RPM1 | Probable disease resistance RPP8-like protein 2 | 2.04 | 3.07 | 9.45 | / | 2.51 | 10.26 |
| 35 | *VIT_15s0045g00980* | K13457 | RPM1, RPS3 | disease resistance protein RPM1 | Putative disease resistance protein At1g50180 | / | / | 2.61 | 1.61 | 2.34 | 1.80 |
| 36 | *VIT_15s0045g00680* | K13457 | RPM1, RPS3 | disease resistance protein RPM1 | Putative disease resistance protein At1g50180 | / | / | 2.48 | 1.76 | 1.51 | 1.77 |
| 37 | *VIT_00s0515g00020* | K13457 | RPM1, RPS3 | disease resistance protein RPM1 | Probable disease resistance RPP8-like protein 4 | / | 1.07 | 2.38 | / | 1.48 | 1.76 |
| 38 | *VIT_15s0045g00920* | K13457 | RPM1, RPS3 | disease resistance protein RPM1 | Putative disease resistance protein At1g50180 | / | 1.83 | 3.67 | / | 2.35 | 2.87 |
| 39 | *VIT_15s0045g01020* | K13457 | RPM1, RPS3 | disease resistance protein RPM1 | Probable disease resistance RPP8-like protein 4 | / | 1.16 | 3.08 | / | 1.62 | 2.10 |
| 40 | *VIT_12s0057g01130* | K13457 | RPM1, RPS3 | disease resistance protein RPM1 | Disease resistance protein RPM1 | / | 1.37 | 2.88 | / | 2.19 | 2.04 |
| 41 | *VIT_06s0009g01360* | K13457 | RPM1, RPS3 | disease resistance protein RPM1 | Disease resistance protein RPM1 | / | / | / | / | / | -1.13 |
| 42 | *VIT_06s0009g01350* | K13457 | RPM1, RPS3 | disease resistance protein RPM1 | Disease resistance protein RPM1 | / | / | / | / | -1.20 | -1.12 |
| 43 | *VIT_07s0005g06200* | K13457 | RPM1, RPS3 | disease resistance protein RPM1 | Putative disease resistance RPP13-like protein 3 | / | / | / | / | / | -1.20 |
| 44 | *VIT_15s0046g02820* | K13457 | RPM1, RPS3 | disease resistance protein RPM1 | Probable disease resistance protein RXW24L | / | -1.02 | -1.04 | / | / | -1.01 |
| 45 | *VIT_15s0046g02750* | K13457 | RPM1, RPS3 | disease resistance protein RPM1 | Putative disease resistance protein At1g50180 | / | -1.59 | -1.81 | / | -1.26 | -1.96 |
| 46 | *VIT_03s0038g01610* | K13457 | RPM1, | disease resistance protein | Putative disease resistance | / | / | / | -2.13 | / | / |

| | | | | | | | | | | | |
|---|---|---|---|---|---|---|---|---|---|---|---|
| | | | RPS3 | RPM1 | protein At1g50180 | | | | | | |
| 47 | *VIT_03s0088g00750* | K13449 | PR1 | pathogenesis-related protein 1 | Basic form of pathogenesis-related protein 1 | 1.35 | / | 2.13 | 2.52 | 1.56 | 1.41 |
| 48 | *VIT_03s0088g00690* | K13449 | PR1 | pathogenesis-related protein 1 | Basic form of pathogenesis-related protein 1 | / | 4.53 | 9.41 | / | 5.96 | 11.07 |
| 49 | *VIT_03s0088g00710* | K13449 | PR1 | pathogenesis-related protein 1 | Basic form of pathogenesis-related protein 1 | / | / | / | 1.26 | 1.29 | 3.37 |
| 50 | *VIT_03s0088g00700* | K13449 | PR1 | pathogenesis-related protein 1 | Basic form of pathogenesis-related protein 1 | / | / | 6.25 | / | / | 8.40 |
| 51 | *VIT_03s0097g00700* | K13449 | PR1 | pathogenesis-related protein 1 | Basic form of pathogenesis-related protein 1 | 4.30 | / | / | 2.54 | / | / |
| 52 | *VIT_03s0088g00910* | K13449 | PR1 | pathogenesis-related protein 1 | Basic form of pathogenesis-related protein 1 | -2.82 | -2.91 | / | / | -1.38 | -4.28 |
| 53 | *VIT_03s0088g00810* | K13449 | PR1 | pathogenesis-related protein 1 | Basic form of pathogenesis-related protein 1 | -2.57 | -1.17 | / | / | / | -2.48 |
| 54 | *VIT_03s0088g00780* | K13449 | PR1 | pathogenesis-related protein 1 | Basic form of pathogenesis-related protein 1 | -1.63 | -1.16 | / | / | / | -2.88 |
| 55 | *VIT_09s0002g00270* | K13459 | RPS2 | disease resistance protein RPS2 | Probable disease resistance protein At5g63020 | / | 1.49 | / | / | 1.34 | 1.16 |
| 56 | *VIT_09s0096g00200* | K13459 | RPS2 | disease resistance protein RPS2 | Probable disease resistance protein At1g12290 | / | / | / | / | 1.37 | 1.04 |
| 57 | *VIT_11s0016g01860* | K13459 | RPS2 | disease resistance protein RPS2 | Disease resistance protein RPS2 | / | / | 1.63 | / | / | 1.41 |
| 58 | *VIT_14s0036g00330* | K13459 | RPS2 | disease resistance protein RPS2 | Disease resistance protein At4g27190 | / | -1.10 | -1.12 | / | / | -1.01 |
| 59 | *VIT_15s0045g00710* | K13459 | RPS2 | disease resistance protein | Probable disease | / | / | / | / | / | -1.08 |

| | | | | | | | | | | | |
|---|---|---|---|---|---|---|---|---|---|---|---|
| | | | | RPS2 | resistance protein At4g27220 | | | | | | |
| 60 | *VIT_19s0014g00900* | K13459 | RPS2 | disease resistance protein RPS2 | Probable disease resistance protein At4g27220 | / | / | / | / | / | -1.32 |
| 61 | *VIT_19s0090g00260* | K13459 | RPS2 | disease resistance protein RPS2 | Disease resistance protein At4g27190 | / | / | / | -1.20 | / | / |
| 62 | *VIT_19s0090g00240* | K13459 | RPS2 | disease resistance protein RPS2 | Disease resistance protein At4g27190 | -1.51 | / | -1.12 | / | / | -1.61 |
| 63 | *VIT_19s0027g01740* | K13459 | RPS2 | disease resistance protein RPS2 | Probable disease resistance protein At5g63020 | / | -1.14 | -1.41 | / | / | -2.12 |
| 64 | *VIT_14s0036g01110* | K13459 | RPS2 | disease resistance protein RPS2 | Probable disease resistance protein At4g27220 | / | / | -2.10 | / | / | -1.87 |
| 65 | *VIT_09s0002g05040* | K13459 | RPS2 | disease resistance protein RPS2 | Disease resistance protein SUMM2 | / | / | -1.71 | / | / | -1.74 |
| 66 | *VIT_14s0036g00010* | K13459 | RPS2 | disease resistance protein RPS2 | Disease resistance protein At4g27190 | / | / | / | -1.29 | / | -1.08 |
| 67 | *VIT_19s0027g01780* | K13459 | RPS2 | disease resistance protein RPS2 | Disease resistance protein RPS5 | / | -1.46 | -1.46 | / | -1.13 | -1.66 |
| 68 | *VIT_11s0052g00270* | K13459 | RPS2 | disease resistance protein RPS2 | Probable disease resistance protein At4g27220 | / | / | -1.80 | / | -1.47 | -2.19 |
| 69 | *VIT_10s0042g00460* | K13459 | RPS2 | disease resistance protein RPS2 | Probable disease resistance protein At4g27220 | / | / | -1.05 | / | / | -1.13 |
| 70 | *VIT_19s0027g01750* | K13459 | RPS2 | disease resistance protein RPS2 | Disease resistance protein SUMM2 | / | -1.46 | / | / | / | / |
| 71 | *VIT_14s0030g00130* | K13459 | RPS2 | disease resistance protein RPS2 | Disease resistance protein At4g27190 | / | -1.71 | -1.24 | / | -1.45 | -2.00 |
| 72 | *VIT_17s0000g07420* | K18875 | EDS1 | enhanced disease susceptibility 1 protein | Protein EDS1 | / | / | 2.36 | / | 1.65 | 1.46 |
| 73 | *VIT_17s0000g07400* | K18875 | EDS1 | enhanced disease susceptibility 1 protein | Protein EDS1L | / | / | 2.00 | / | 1.55 | 1.30 |
| 74 | *VIT_17s0000g07560* | K18875 | EDS1 | enhanced disease | Protein EDS1L | / | / | 1.32 | / | / | 1.44 |

| | | | | | | | | | | | |
|---|---|---|---|---|---|---|---|---|---|---|---|
| | | | | susceptibility 1 protein | | | | | | | |
| 75 | *VIT_17s0000g07370* | K18875 | EDS1 | enhanced disease susceptibility 1 protein | Protein EDS1 | / | / | 2.20 | / | 1.44 | 1.46 |
| 76 | *VIT_05s0062g00690* | K04079 | HSP90A, htpG | heat shock protein 90kDa beta | Heat shock protein 81-3 | / | / | 4.45 | 3.45 | / | 6.76 |
| 77 | *VIT_02s0025g00280* | K04079 | HSP90A, htpG | heat shock protein 90kDa beta | Heat shock protein 83 | 3.78 | / | / | / | / | / |
| 78 | *VIT_16s0050g01150* | K04079 | HSP90A, htpG | heat shock protein 90kDa beta | Heat shock protein 83 | 3.94 | / | / | 2.71 | / | / |
| 79 | *VIT_01s0010g00680* | K09487 | HSP90B, TRA1 | heat shock protein 90kDa beta | Heat shock protein 90-5, chloroplastic | / | / | / | / | / | -1.50 |
| 80 | *VIT_18s0001g14500* | K09487 | HSP90B, TRA1 | heat shock protein 90kDa beta | Endoplasmin homolog | / | / | 1.12 | / | / | 1.84 |
| 81 | *VIT_13s0047g00260* | K13412 | CPK | calcium-dependent protein kinase [EC:2.7.11.1] | Calcium and calcium/calmodulin-dependent serine/threonine-protein kinase DMI-3 | / | 1.78 | 3.59 | / | 2.34 | 3.72 |
| 82 | *VIT_08s0007g08300* | K13412 | CPK | calcium-dependent protein kinase [EC:2.7.11.1] | Calcium-dependent protein kinase 32 | / | / | 1.73 | / | / | 1.20 |
| 83 | *VIT_06s0004g02300* | K13412 | CPK | calcium-dependent protein kinase [EC:2.7.11.1] | Calcium-dependent protein kinase 8 | 3.48 | / | / | 2.78 | / | / |
| 84 | *VIT_19s0090g00410* | K13412 | CPK | calcium-dependent protein kinase [EC:2.7.11.1] | Calcium-dependent protein kinase 19 | / | / | 1.40 | / | / | 1.60 |
| 85 | *VIT_03s0038g03960* | K13412 | CPK | calcium-dependent protein kinase [EC:2.7.11.1] | Calcium-dependent protein kinase 4 | / | / | 1.29 | / | / | / |
| 86 | *VIT_04s0023g03420* | K13412 | CPK | calcium-dependent protein kinase [EC:2.7.11.1] | Calcium-dependent protein kinase 28 | / | / | 1.18 | / | / | / |
| 87 | *VIT_12s0028g02280* | K13412 | CPK | calcium-dependent protein kinase [EC:2.7.11.1] | Calcium-dependent protein kinase 32 | -2.16 | / | / | -1.69 | / | -5.14 |
| 88 | *VIT_09s0002g07640* | K00864 | glpK, GK | glycerol kinase [EC:2.7.1.30] | Glycerol kinase | / | / | 1.04 | / | / | 1.11 |
| 89 | *VIT_09s0002g07860* | K00864 | glpK, GK | glycerol kinase [EC:2.7.1.30] | Glycerol kinase | / | / | / | / | / | 1.02 |
| 90 | *VIT_14s0108g01000* | K02183 | CALM | calmodulin | Probable calcium-binding protein CML45 | / | 1.01 | 1.17 | 1.10 | 1.68 | / |
| 91 | *VIT_05s0077g00810* | K02183 | CALM | calmodulin | Calcium-binding protein CP1 | 1.90 | / | / | 2.64 | / | / |

| | | | | | | | | | | | |
|---|---|---|---|---|---|---|---|---|---|---|---|
| 92 | *VIT_05s0077g00190* | K02183 | CALM | calmodulin | Calcium-binding protein CP1 | 1.65 | / | / | 2.42 | / | / |
| 93 | *VIT_01s0026g02590* | K02183 | CALM | calmodulin | Probable calcium-binding protein CML30 | / | / | / | 4.95 | / | / |
| 94 | *VIT_12s0142g00700* | K13414 | MEKK1 | mitogen-activated protein kinase kinase kinase 1 [EC:2.7.11.25] | Mitogen-activated protein kinase kinase kinase 1 | -1.23 | / | -1.33 | / | / | -1.41 |
| 95 | *VIT_12s0142g00690* | K13414 | MEKK1 | mitogen-activated protein kinase kinase kinase 1 [EC:2.7.11.25] | Mitogen-activated protein kinase kinase kinase 1 | / | / | -1.05 | / | / | -1.51 |
| 96 | *VIT_08s0058g00690* | K13424 | WRKY33 | WRKY transcription factor 33 | Probable WRKY transcription factor 33 | 2.04 | 2.79 | 3.44 | 2.66 | 3.10 | 2.24 |
| 97 | *VIT_06s0004g07500* | K13424 | WRKY33 | WRKY transcription factor 33 | WRKY transcription factor WRKY24 | / | 1.38 | 2.09 | / | 1.46 | 1.98 |
| 98 | *VIT_02s0025g00420* | K13425 | WRKY22 | WRKY transcription factor 22 | WRKY transcription factor 22 | 2.40 | 3.98 | 4.41 | 2.31 | 4.48 | 3.98 |
| 99 | *VIT_15s0046g02190* | K13425 | WRKY22 | WRKY transcription factor 22 | WRKY transcription factor 22 | 1.45 | 2.53 | 2.04 | 1.16 | 2.48 | 1.74 |
| 100 | *VIT_04s0023g00470* | K18835 | WRKY2 | WRKY transcription factor 2 | Probable WRKY transcription factor 2 | / | / | -1.07 | / | / | -1.48 |
| 101 | *VIT_17s0053g00860* | K05391 | CNGC | cyclic nucleotide gated channel, plant | Probable cyclic nucleotide-gated ion channel 20, chloroplastic | / | 1.74 | / | / | / | / |
| 102 | *VIT_15s0046g00980* | K05391 | CNGC | cyclic nucleotide gated channel, plant | Cyclic nucleotide-gated ion channel 1 | / | / | / | / | / | -1.06 |
| 103 | *VIT_10s0003g02910* | K13420 | FLS2 | LRR receptor-like serine/threonine-protein kinase FLS2 [EC:2.7.11.1] | LRR receptor-like serine/threonine-protein kinase FLS2 | / | / | / | / | 1.39 | / |
| 104 | *VIT_12s0142g00640* | K13420 | FLS2 | LRR receptor-like serine/threonine-protein kinase FLS2 [EC:2.7.11.1] | LRR receptor-like serine/threonine-protein kinase FLS2 | / | / | -1.25 | / | / | -1.48 |
| 105 | *VIT_09s0002g08640* | K13420 | FLS2 | LRR receptor-like serine/threonine-protein kinase FLS2 [EC:2.7.11.1] | Probable LRR receptor-like serine/threonine-protein kinase | / | / | / | / | / | -1.10 |
| 106 | *VIT_01s0150g00440* | K13447 | RBOH | respiratory burst oxidase | Respiratory burst oxidase | 1.96 | / | 2.31 | 3.20 | / | 2.11 |

| | | | | | | | | | | | |
|---|---|---|---|---|---|---|---|---|---|---|---|
| | | | | [EC:1.6.3.- 1.11.1.-] | homolog protein D | | | | | | |
| 107 | *VIT_02s0025g00510* | K13447 | RBOH | respiratory burst oxidase [EC:1.6.3.- 1.11.1.-] | Respiratory burst oxidase homolog protein A | / | 1.32 | 1.76 | / | 1.43 | 1.92 |
| 108 | *VIT_19s0014g02830* | K13447 | RBOH | respiratory burst oxidase [EC:1.6.3.- 1.11.1.-] | Respiratory burst oxidase homolog protein C | / | -1.03 | / | / | / | / |
| 109 | *VIT_05s0029g00690* | K13456 | RIN4 | RPM1-interacting protein 4 | RPM1-interacting protein 4 | / | / | 1.73 | 1.12 | / | 1.86 |
| 110 | *VIT_00s0516g00010* | K13456 | RIN4 | RPM1-interacting protein 4 | NOI-like protein | / | / | / | 1.46 | / | / |
| 111 | *VIT_00s0231g00040* | K13456 | RIN4 | RPM1-interacting protein 4 | RPM1-interacting protein 4 | / | / | / | / | / | 1.47 |
| 112 | *VIT_14s0068g01540* | K13430 | PBS1 | serine/threonine-protein kinase PBS1 [EC:2.7.11.1] | Serine/threonine-protein kinase PBS1 | / | / | / | / | / | 1.44 |
| 113 | *VIT_01s0146g00250* | K13430 | PBS1 | serine/threonine-protein kinase PBS1 [EC:2.7.11.1] | Serine/threonine-protein kinase PBS1 | / | / | 1.11 | / | / | / |
| 114 | *VIT_08s0032g01220* | K13412 | CPK29 | calcium-dependent protein kinase [EC:2.7.11.1] | Calcium-dependent protein kinase 1 | 1.01 | 2.46 | 4.33 | 2.48 | 3.29 | 2.92 |
| 115 | *VIT_18s0001g00990* | K13412 | CPK29 | calcium-dependent protein kinase [EC:2.7.11.1] | Calcium-dependent protein kinase 29 | / | / | / | / | / | -1.26 |
| 116 | *VIT_14s0060g02320* | K13447 | ATRBOH B | respiratory burst oxidase [EC:1.6.3.- 1.11.1.-] | Respiratory burst oxidase homolog protein B | 2.81 | 2.48 | 5.12 | 2.93 | 3.29 | 6.31 |
| 117 | *VIT_05s0020g04940* | K13457 | RPM1, RPS3 | disease resistance protein RPM1 | unnamed protein product | 1.73 | / | / | 1.31 | / | / |
| 118 | *VIT_18s0089g00630* | K13470 | CF4 | disease resistance protein | Receptor-like protein Cf-9 | / | / | 3.54 | / | / | 3.62 |
| 119 | *VIT_11s0016g02970* | K04368 | MAP2K1 , MEK1 | mitogen-activated protein kinase kinase 1 [EC:2.7.12.2] | Mitogen-activated protein kinase kinase 6 | / | / | / | / | / | 1.51 |
| 120 | *VIT_06s0004g08190* | K13434 | PTI6 | Pathogenesis-related genes transcriptional activator PTI6 | pathogenesis-related genes transcriptional activator PTI6 | / | -1.07 | / | / | / | / |

**Table S8.** DEGs involved in the plant hormone signal transduction pathway of the tissue cultured seedlings in response to endophytes fungi Epi R2-21 and Alt XHYN2 inoculation (|$Log_2FC$|≥1; *p*-adjust <0.05). DEGs were analyzed in six pairwise comparisons, R2-21_6h vs. Con_6h comparison, R2-21_6d vs. Con_6d, R2-21_15d vs. Con_15d, XHYN2_6h vs. Con_6h, XHYN2_6d vs. Con_6d, XHYN2_15d vs. Con_15d. R2-21_6h, R2-21_6d, and R2-21_15d represent leaf samples from Epi R2-21 treatments collected at 6 h, 6 d, and 15 d after inoculation, respectively; XHYN2_6h, XHYN2_6d, and XHYN2_15d represent leaf samples from Alt XHYN2 treatments collected at 6 h, 6 d, and 15 d after inoculation, respectively; and Con_6h, Con_6d, and Con_15d represent leaf samples from the control collected at 6 h, 6 d, and 15 d after inoculation, respectively. "/"represents no DEG enriched significantly in the pairwise comparison.

| No. | gene ID | KO ID | Gene product | KEGG definition | Gene description | $Log_2FC$ | | | | | |
|---|---|---|---|---|---|---|---|---|---|---|---|
| | | | | | | **R2-21_6h vs. Con_6h** | **R2-21_6d vs. Con_6d** | **R2-21_15d vs. Con_15d** | **XHYN2_6h vs. Con_6h** | **XHYN2_6d vs. Con_6d** | **XHYN2_15d vs. Con_15d** |
| 1 | *VIT_07s0129g00660* | K14487 | GH3 | auxin responsive GH3 gene family | Probable indole-3-acetic acid-amido synthetase GH3.1 | 2.96 | 4.10 | 8.28 | 3.81 | 3.17 | 8.20 |
| 2 | *VIT_03s0091g00310* | K14487 | GH3 | auxin responsive GH3 gene family | Probable indole-3-acetic acid-amido synthetase GH3.1 | 1.47 | 3.19 | 7.20 | 2.60 | 2.82 | 8.40 |
| 3 | *VIT_19s0014g04690* | K14487 | GH3 | auxin responsive GH3 gene family | Indole-3-acetic acid-amido synthetase GH3.6 | / | / | 6.14 | / | 2.71 | 7.74 |
| 4 | *VIT_07s0104g00800* | K14487 | GH3 | auxin responsive GH3 gene family | Probable indole-3-acetic acid-amido synthetase GH3.6 | / | / | / | / | 1.00 | 1.20 |
| 5 | *VIT_01s0150g00300* | K14487 | GH3 | auxin responsive GH3 gene family | Indole-3-acetic acid-amido synthetase GH3.17 | / | -3.06 | -2.73 | / | -3.37 | / |
| 6 | *VIT_07s0005g00090* | K14487 | GH3 | auxin responsive GH3 gene family | Putative indole-3-acetic acid-amido synthetase GH3.9 | / | / | / | / | -2.78 | / |
| 7 | *VIT_15s0048g00530* | K14488 | SAUR | SAUR family protein | Auxin-responsive protein SAUR36 | 1.04 | 1.58 | 2.25 | 1.01 | 2.19 | 1.94 |
| 8 | *VIT_08s0058g01160* | K14488 | SAUR | SAUR family protein | indole-3-acetic acid-induced protein ARG7-like | 1.81 | 2.13 | 3.20 | / | 3.23 | 2.66 |
| 9 | *VIT_09s0002g00670* | K14488 | SAUR | SAUR family protein | Auxin-responsive protein | 2.00 | 2.08 | 2.39 | / | 2.21 | 3.16 |

| | | | | | | | | | | | |
|---|---|---|---|---|---|---|---|---|---|---|---|
| | | | | | SAUR71 | | | | | | |
| 10 | *VIT_16s0098g01150* | K14488 | SAUR | SAUR family protein | Auxin-responsive protein SAUR32 | 1.48 | 1.73 | 2.01 | / | 1.81 | 2.15 |
| 11 | *VIT_11s0016g00520* | K14488 | SAUR | SAUR family protein | Auxin-induced protein 15A | / | 2.11 | 4.33 | / | 1.97 | 5.33 |
| 12 | *VIT_19s0085g00010* | K14488 | SAUR | SAUR family protein | Auxin-responsive protein SAUR72 | / | 1.66 | 1.92 | / | 1.15 | 1.67 |
| 13 | *VIT_12s0028g00690* | K14488 | SAUR | SAUR family protein | Auxin-responsive protein SAUR71 | / | 2.29 | 2.08 | / | 2.63 | 2.52 |
| 14 | *VIT_03s0038g01310* | K14488 | SAUR | SAUR family protein | Auxin-responsive protein SAUR23 | / | / | / | / | / | 1.75 |
| 15 | *VIT_04s0023g00580* | K14488 | SAUR | SAUR family protein | Auxin-induced protein X10A | / | / | / | / | 1.02 | / |
| 16 | *VIT_02s0154g00010* | K14488 | SAUR | SAUR family protein | Auxin-responsive protein SAUR36 | / | / | / | / | 1.05 | / |
| 17 | *VIT_01s0146g00210* | K14488 | SAUR | SAUR family protein | auxin-responsive protein SAUR71 | / | / | / | / | 2.60 | / |
| 18 | *VIT_08s0040g01670* | K14488 | SAUR | SAUR family protein | Auxin-responsive protein SAUR50 | / | / | 4.90 | / | / | 4.86 |
| 19 | *VIT_18s0001g13960* | K14488 | SAUR | SAUR family protein | Auxin-responsive protein SAUR50 | / | / | 2.04 | / | / | 2.48 |
| 20 | *VIT_01s0146g00180* | K14488 | SAUR | SAUR family protein | Auxin-responsive protein SAUR32 | -1.04 | / | 1.10 | / | 1.48 | 1.37 |
| 21 | *VIT_04s0023g00530* | K14488 | SAUR | SAUR family protein | Auxin-responsive protein SAUR23 | / | -3.55 | / | / | -3.41 | / |
| 22 | *VIT_03s0038g01090* | K14488 | SAUR | SAUR family protein | Auxin-responsive protein SAUR21 | / | -3.70 | / | / | -5.95 | / |
| 23 | *VIT_04s0023g03230* | K14488 | SAUR | SAUR family protein | Auxin-responsive protein SAUR50 | / | -1.31 | -1.44 | / | -1.55 | / |
| 24 | *VIT_03s0038g01270* | K14488 | SAUR | SAUR family protein | Auxin-responsive protein SAUR21 | / | -6.26 | / | / | -6.10 | / |
| 25 | *VIT_03s0038g00950* | K14488 | SAUR | SAUR family protein | Auxin-responsive protein SAUR50 | / | -1.72 | -3.67 | / | -2.84 | -1.85 |
| 26 | *VIT_03s0038g01110* | K14488 | SAUR | SAUR family protein | Auxin-responsive protein SAUR20 | / | -3.77 | -5.44 | / | -6.10 | / |
| 27 | *VIT_03s0038g01130* | K14488 | SAUR | SAUR family protein | Auxin-responsive protein | / | -3.64 | -5.85 | / | -5.24 | / |

| | | | | | | | | | | | |
|---|---|---|---|---|---|---|---|---|---|---|---|
| | | | | | SAUR20 | | | | | | |
| 28 | *VIT_03s0038g01150* | K14488 | SAUR | SAUR family protein | Auxin-responsive protein SAUR21 | / | -2.66 | -4.29 | / | -4.31 | -2.94 |
| 29 | *VIT_03s0038g01190* | K14488 | SAUR | SAUR family protein | Auxin-responsive protein SAUR23 | / | -5.56 | / | / | / | / |
| 30 | *VIT_11s0016g04490* | K14484 | IAA | auxin-responsive protein IAA | Auxin-responsive protein IAA17 | / | 1.56 | 2.42 | / | 1.94 | 1.72 |
| 31 | *VIT_05s0020g04680* | K14484 | IAA | auxin-responsive protein IAA | Auxin-induced protein 22D | 1.01 | / | / | 2.64 | / | / |
| 32 | *VIT_09s0002g05160* | K14484 | IAA | auxin-responsive protein IAA | Auxin-responsive protein IAA17 | 2.20 | / | / | 2.29 | / | / |
| 33 | *VIT_09s0002g05150* | K14484 | IAA | auxin-responsive protein IAA | Auxin-induced protein 22A | 1.70 | -2.58 | / | 2.90 | -2.94 | / |
| 34 | *VIT_14s0030g02310* | K14484 | IAA | auxin-responsive protein IAA | Auxin-induced protein 22D | / | -1.84 | -2.35 | 1.85 | -2.36 | -1.62 |
| 35 | *VIT_07s0005g04380* | K14484 | IAA | auxin-responsive protein IAA | Auxin-responsive protein IAA12 | / | -1.27 | / | 1.41 | -1.40 | / |
| 36 | *VIT_14s0081g00010* | K14484 | IAA | auxin-responsive protein IAA | Auxin-responsive protein IAA16 | / | -1.57 | -1.29 | 1.56 | -2.19 | -1.77 |
| 37 | *VIT_04s0008g00220* | K14484 | IAA | auxin-responsive protein IAA | Auxin-responsive protein IAA26 | -1.09 | / | / | -1.28 | / | / |
| 38 | *VIT_07s0141g00270* | K14484 | IAA | auxin-responsive protein IAA | Auxin-induced protein 22D | / | -1.74 | -1.04 | 2.19 | -2.06 | / |
| 39 | *VIT_11s0016g05640* | K14484 | IAA | auxin-responsive protein IAA | auxin-responsive protein IAA9 | / | / | / | / | -1.77 | / |
| 40 | *VIT_05s0020g01070* | K14484 | IAA | auxin-responsive protein IAA | auxin-responsive protein IAA31 | / | -5.62 | / | / | / | -4.94 |
| 41 | *VIT_04s0008g05560* | K14484 | IAA | auxin-responsive protein IAA | Auxin-responsive protein IAA29 | / | -2.62 | -1.70 | / | -2.62 | -1.52 |
| 42 | *VIT_05s0020g04690* | K14484 | IAA | auxin-responsive protein IAA | Auxin-responsive protein IAA7 | / | -1.70 | / | / | -1.71 | -1.96 |
| 43 | *VIT_07s0141g00290* | K14484 | IAA | auxin-responsive protein IAA | Auxin-responsive protein IAA16 | / | -2.09 | -1.68 | / | -2.45 | -2.82 |
| 44 | *VIT_09s0002g04080* | K14484 | IAA | auxin-responsive protein IAA | Auxin-responsive protein IAA27 | / | -1.80 | -1.72 | / | -2.22 | -2.79 |
| 45 | *VIT_11s0016g03540* | K14484 | IAA | auxin-responsive protein IAA | Auxin-responsive protein | / | -1.87 | -1.78 | / | -1.86 | -1.88 |

| | | | | | | | | | | | |
|---|---|---|---|---|---|---|---|---|---|---|---|
| | | | | | IAA27 | | | | | | |
| 46 | *VIT_05s0049g01970* | K14484 | IAA | auxin-responsive protein IAA | Auxin-responsive protein IAA13 | / | / | / | / | -1.06 | -1.40 |
| 47 | *VIT_18s0001g03540* | K13946 | AUX1 | auxin influx carrier (AUX1 LAX family) | Auxin transporter-like protein 3 | / | -1.10 | / | / | / | / |
| 48 | *VIT_03s0038g02140* | K13946 | AUX1 | auxin influx carrier (AUX1 LAX family) | Auxin transporter-like protein 5 | / | -1.46 | / | / | -1.57 | -2.25 |
| 49 | *VIT_08s0007g02030* | K13946 | AUX1 | auxin influx carrier (AUX1 LAX family) | Auxin transporter-like protein 2 | / | -1.11 | -1.39 | / | -1.43 | -1.77 |
| 50 | *VIT_07s0104g01230* | K14486 | ARF | auxin response factor | Auxin response factor 2 | 1.17 | 3.66 | 2.75 | / | 2.82 | 3.21 |
| 51 | *VIT_18s0001g13930* | K14486 | ARF | auxin response factor | Auxin response factor 5 | / | / | / | / | / | 1.25 |
| 52 | *VIT_10s0003g00420* | K14486 | ARF | auxin response factor | Auxin response factor 3 | / | -1.13 | -1.04 | / | -1.00 | -1.91 |
| 53 | *VIT_15s0046g00290* | K14486 | ARF | auxin response factor | Auxin response factor 7 | 1.06 | / | -1.33 | / | -1.03 | -1.50 |
| 54 | *VIT_02s0025g01740* | K14486 | ARF | auxin response factor | Auxin response factor 7 | / | / | / | / | -1.42 | -1.09 |
| 55 | *VIT_11s0052g01210* | K14504 | TCH4 | xyloglucan:xyloglucosyl transferase TCH4 [EC:2.4.1.207] | Probable xyloglucan endotransglucosylase/hydrolase protein 23 | / | / | 2.76 | / | / | / |
| 56 | *VIT_11s0052g01330* | K14504 | TCH4 | xyloglucan:xyloglucosyl transferase TCH4 [EC:2.4.1.207] | Probable xyloglucan endotransglucosylase/hydrolase protein 23 | / | / | 3.10 | / | / | 2.00 |
| 57 | *VIT_11s0052g01310* | K14504 | TCH4 | xyloglucan:xyloglucosyl transferase TCH4 [EC:2.4.1.207] | Xyloglucan endotransglucosylase/hydrolase 2 | / | / | 2.68 | / | 2.24 | / |
| 58 | *VIT_11s0052g01220* | K14504 | TCH4 | xyloglucan:xyloglucosyl transferase TCH4 [EC:2.4.1.207] | Probable xyloglucan endotransglucosylase/hydrolase protein 23 | / | 1.19 | 1.60 | / | 1.57 | / |
| 59 | *VIT_11s0052g01340* | K14504 | TCH4 | xyloglucan:xyloglucosyl transferase TCH4 [EC:2.4.1.207] | Xyloglucan endotransglucosylase/hydrolase 2 | / | 1.36 | 2.16 | / | 2.09 | / |
| 60 | *VIT_11s0052g01180* | K14504 | TCH4 | xyloglucan:xyloglucosyl transferase TCH4 [EC:2.4.1.207] | Probable xyloglucan endotransglucosylase/hydrolase protein 23 | / | 1.59 | 1.69 | / | 2.47 | / |
| 61 | *VIT_11s0052g01190* | K14504 | TCH4 | xyloglucan:xyloglucosyl transferase TCH4 [EC:2.4.1.207] | Probable xyloglucan endotransglucosylase/hydrolase protein 23 | / | 1.27 | 1.83 | / | 1.92 | / |
| 62 | *VIT_11s0052g01280* | K14504 | TCH4 | xyloglucan:xyloglucosyl | Probable xyloglucan | / | / | 4.78 | / | / | 3.74 |

| | | | | | | | | | | | |
|---|---|---|---|---|---|---|---|---|---|---|---|
| | | | | transferase TCH4 [EC:2.4.1.207] | endotransglucosylase/hydrolase protein 23 | | | | | | |
| 63 | *VIT_11s0052g01200* | K14504 | TCH4 | xyloglucan:xyloglucosyl transferase TCH4 [EC:2.4.1.207] | Probable xyloglucan endotransglucosylase/hydrolase protein 23 | / | / | 1.25 | / | 1.77 | / |
| 64 | *VIT_11s0052g01250* | K14504 | TCH4 | xyloglucan:xyloglucosyl transferase TCH4 [EC:2.4.1.207] | Xyloglucan endotransglucosylase/hydrolase 2 | / | / | / | -1.11 | / | / |
| 65 | *VIT_03s0088g00710* | K13449 | PR1 | pathogenesis-related protein 1 | Basic form of pathogenesis-related protein 1 | / | / | / | / | 1.29 | / |
| 66 | *VIT_03s0088g00690* | K13449 | PR1 | pathogenesis-related protein 1 | Basic form of pathogenesis-related protein 1 | / | 4.53 | 9.41 | / | 5.96 | 11.07 |
| 67 | *VIT_03s0088g00750* | K13449 | PR1 | pathogenesis-related protein 1 | Basic form of pathogenesis-related protein 1 | / | / | 2.13 | 2.52 | 1.56 | / |
| 68 | *VIT_03s0097g00700* | K13449 | PR1 | pathogenesis-related protein 1 | Basic form of pathogenesis-related protein 1 | 4.30 | / | / | 2.54 | / | / |
| 69 | *VIT_03s0088g00700* | K13449 | PR1 | pathogenesis-related protein 1 | Basic form of pathogenesis-related protein 1 | / | / | 6.25 | / | / | 8.40 |
| 70 | *VIT_03s0088g00780* | K13449 | PR1 | pathogenesis-related protein 1 | Basic form of pathogenesis-related protein 1 | -1.63 | -1.16 | / | / | / | -2.88 |
| 71 | *VIT_03s0088g00810* | K13449 | PR1 | pathogenesis-related protein 1 | Basic form of pathogenesis-related protein 1 | / | / | / | / | / | -2.48 |
| 72 | *VIT_01s0146g00480* | K13464 | JAZ | jasmonate ZIM domain-containing protein | Protein TIFY 9 | / | 3.03 | 7.27 | / | 3.18 | 8.84 |
| 73 | *VIT_11s0016g00710* | K13464 | JAZ | jasmonate ZIM domain-containing protein | Protein TIFY 10A | / | 1.92 | 1.95 | / | 2.35 | 2.26 |
| 74 | *VIT_09s0002g00890* | K13464 | JAZ | jasmonate ZIM domain-containing protein | Protein TIFY 10A | / | 1.79 | 1.85 | / | 1.77 | 3.04 |
| 75 | *VIT_01s0011g05560* | K13464 | JAZ | jasmonate ZIM | Protein TIFY 6B | / | / | / | / | / | 1.24 |

| | | | | | | | | | | | |
|---|---|---|---|---|---|---|---|---|---|---|---|
| | | | | domain-containing protein | | | | | | | |
| 76 | *VIT_17s0000g02230* | K13464 | JAZ | jasmonate ZIM domain-containing protein | Protein TIFY 6B | / | -1.61 | -1.16 | / | -1.55 | -1.54 |
| 77 | *VIT_11s0016g03180* | K14497 | PP2C | protein phosphatase 2C [EC:3.1.3.16] | Probable protein phosphatase 2C 6 | 2.40 | / | / | / | / | / |
| 78 | *VIT_13s0019g02200* | K14497 | PP2C | protein phosphatase 2C [EC:3.1.3.16] | Protein phosphatase 2C 37 | / | / | / | / | / | 1.20 |
| 79 | *VIT_09s0002g03600* | K14497 | PP2C | protein phosphatase 2C [EC:3.1.3.16] | Protein phosphatase 2C 56 | / | / | / | / | / | -1.17 |
| 80 | *VIT_06s0004g05460* | K14497 | PP2C | protein phosphatase 2C [EC:3.1.3.16] | Probable protein phosphatase 2C 78 | / | / | / | / | -1.29 | / |
| 81 | *VIT_11s0016g03170* | K14497 | PP2C | protein phosphatase 2C [EC:3.1.3.16] | Pseudo histidine-containing phosphotransfer protein 2 | / | / | / | / | -1.27 | / |
| 82 | *VIT_04s0008g01420* | K14497 | PP2C | protein phosphatase 2C [EC:3.1.3.16] | Protein phosphatase 2C 16 | / | / | / | / | / | -1.13 |
| 83 | *VIT_05s0049g00510* | K14516 | ERF1 | ethylene-responsive transcription factor 1 | Ethylene-responsive transcription factor 1B | / | / | 1.80 | / | / | 2.97 |
| 84 | *VIT_14s0081g00730* | K14516 | ERF1 | ethylene-responsive transcription factor 1 | Ethylene-responsive transcription factor 1B | / | / | 1.03 | / | / | / |
| 85 | *VIT_07s0005g03230* | K14516 | ERF1 | ethylene-responsive transcription factor 1 | Ethylene-responsive transcription factor 1B | / | / | / | / | / | 6.48 |
| 86 | *VIT_07s0005g03260* | K14516 | ERF1 | ethylene-responsive transcription factor 1 | Ethylene-responsive transcription factor 1B | -1.42 | / | / | / | / | / |
| 87 | *VIT_13s0067g03070* | K14492 | ARR-A | two-component response regulator ARR-A family | Two-component response regulator ARR17 | / | 1.82 | 2.44 | / | 2.04 | 2.65 |
| 88 | *VIT_01s0026g00940* | K14492 | ARR-A | two-component response regulator ARR-A family | Two-component response regulator ARR4 | / | / | 1.84 | / | / | / |
| 89 | *VIT_18s0001g02540* | K14492 | ARR-A | two-component response regulator ARR-A family | Two-component response regulator ORR4 | / | / | / | / | / | -4.21 |
| 90 | *VIT_02s0012g01270* | K14496 | PYL | abscisic acid receptor PYR/PYL family | Abscisic acid receptor PYL1 | / | 1.12 | 1.15 | / | 1.65 | 1.57 |
| 91 | *VIT_08s0058g00470* | K14496 | PYL | abscisic acid receptor PYR/PYL family | Abscisic acid receptor PYL4 | / | / | / | / | 1.36 | / |
| 92 | *VIT_13s0067g01940* | K14496 | PYL | abscisic acid receptor PYR/PYL family | Abscisic acid receptor PYL4 | / | 1.40 | / | / | 2.00 | / |

| | | | | | | | | | | | |
|---|---|---|---|---|---|---|---|---|---|---|---|
| 93 | *VIT_04s0008g00210* | K14490 | AHP | histidine-containing phosphotransfer peotein | Pseudo histidine-containing phosphotransfer protein 5 OS | / | -3.09 | -2.17 | / | -1.96 | -4.17 |
| 94 | *VIT_09s0002g03520* | K14490 | AHP | histidine-containing phosphotransfer peotein | Histidine-containing phosphotransfer protein 4 | / | / | -3.00 | / | -3.99 | -3.35 |
| 95 | *VIT_05s0020g02210* | K14490 | AHP | histidine-containing phosphotransfer peotein | Histidine-containing phosphotransfer protein 1 | / | / | -1.01 | / | -1.21 | -1.52 |
| 96 | *VIT_06s0004g05810* | K14500 | BSK | BR-signaling kinase [EC:2.7.11.1] | Serine/threonine-protein kinase BSK5 | / | / | 1.20 | / | / | 1.98 |
| 97 | *VIT_03s0038g03270* | K14500 | BSK | BR-signaling kinase [EC:2.7.11.1] | Serine/threonine-protein kinase BSK1 | / | / | 1.03 | / | / | 1.31 |
| 98 | *VIT_18s0001g00180* | K14500 | BSK | BR-signaling kinase [EC:2.7.11.1] | Serine/threonine-protein kinase BSK1 | / | / | 1.05 | / | / | 1.28 |
| 99 | *VIT_02s0012g01140* | K14500 | BSK | BR-signaling kinase [EC:2.7.11.1] | Serine/threonine-protein kinase BSK2 | / | -1.01 | / | / | -1.39 | -1.33 |
| 100 | *VIT_18s0001g09920* | K14505 | CYCD3 | cyclin D3, plant | Cyclin-D3-1 | 4.94 | / | / | 6.20 | / | -3.37 |
| 101 | *VIT_07s0129g01100* | K14505 | CYCD3 | cyclin D3, plant | Cyclin-D3-2 | / | / | -1.82 | / | / | -2.34 |
| 102 | *VIT_03s0180g00040* | K14505 | CYCD3 | cyclin D3, plant | Cyclin-D3-2 | / | -1.52 | / | / | / | -2.72 |
| 103 | *VIT_12s0055g00420* | K14432 | ABF | ABA responsive element binding factor | bZIP transcription factor 46 | / | / | 1.17 | / | / | 1.72 |
| 104 | *VIT_03s0063g00310* | K14432 | ABF | ABA responsive element binding factor | ABSCISIC ACID-INSENSITIVE 5-like protein 7 | / | / | -1.07 | / | / | -1.14 |
| 105 | *VIT_14s0030g01240* | K14485 | TIR 1 | transport inhibitor response 1 | Protein TRANSPORT INHIBITOR RESPONSE 1 | / | / | / | / | / | 1.11 |
| 106 | *VIT_07s0104g01320* | K14485 | TIR1 | transport inhibitor response 1 | Protein TRANSPORT INHIBITOR RESPONSE 1 | / | / | -1.08 | / | / | -1.10 |
| 107 | *VIT_18s0001g06310* | K14498 | SNRK2 | serine/threonine-protein kinase SRK2 [EC:2.7.11.1] | Serine/threonine-protein kinase SAPK2 | / | / | 1.45 | / | 1.03 | 1.81 |
| 108 | *VIT_07s0031g03210* | K14498 | SNRK2 | serine/threonine-protein kinase SRK2 [EC:2.7.11.1] | Serine/threonine-protein kinase SAPK7 | / | / | / | / | -1.20 | -1.28 |
| 109 | *VIT_06s0004g05240* | K14509 | ETR, ERS | ethylene receptor [EC:2.7.13.-] | Ethylene receptor 2 | / | 2.84 | / | / | 3.22 | 2.27 |

| | | | | | | | | | | | |
|---|---|---|---|---|---|---|---|---|---|---|---|
| 110 | *VIT_05s0049g00090* | K14509 | ETR, ERS | ethylene receptor [EC:2.7.13.-] | Ethylene receptor 2 | -1.55 | / | / | -1.11 | / | 1.18 |
| 111 | *VIT_07s0104g00930* | K14493 | GID1 | gibberellin receptor GID1 [EC:3.-.-.-] | Gibberellin receptor GID1B | / | 1.10 | 1.52 | / | 1.20 | 2.01 |
| 112 | *VIT_11s0016g05410* | K14515 | EBF1_2 | EIN3-binding F-box protein | EIN3-binding F-box protein 1 | / | / | / | / | / | 1.15 |
| 113 | *VIT_01s0011g04220* | K14491 | ARR-B | two-component response regulator ARR-B family | Myb family transcription factor PHL13 | 3.38 | / | 2.32 | 3.44 | / | 3.50 |
| 114 | *VIT_15s0048g02820* | K13422 | MYC2 | transcription factor MYC2 | Transcription factor bHLH14 | / | -2.29 | -1.88 | / | -2.13 | / |
| 115 | *VIT_08s0007g05740* | K14508 | NPR1 | regulatory protein NPR1 | Regulatory protein NPR5 | / | / | / | / | / | -1.05 |
| 116 | *VIT_01s0011g06190* | K14489 | CRE1 | arabidopsis histidine kinase 2/3/4 (cytokinin receptor) [EC:2.7.13.3] | Histidine kinase 4 | / | / | / | / | / | -2.13 |
| 117 | *VIT_07s0005g05100* | K16189 | PIF4 | phytochrome-interacting factor 4 | Transcription factor PIF1 | / | / | / | / | / | -1.04 |
| 118 | *VIT_07s0005g02510* | K12126 | PIF3 | phytochrome-interacting factor 3 | Transcription factor APG | / | -1.40 | -1.55 | / | -2.36 | -1.81 |
| 119 | *VIT_08s0007g06160* | K14431 | TGA | transcription factor TGA | bZIP transcription factor TGA10 | 1.99 | 3.96 | 4.07 | 2.40 | 3.36 | 4.38 |
