# Supplementary figures and images for: The Interactions between Two Fungal Endophytes Epicoccum layuense R2-21 and Alternaria alternata XHYN2 and Grapevines (Vitis vinifera) with De Novo Established Symbionts under Aseptic Conditions
Source: J Fungi (Basel). 2023 Nov 30;9(12):1154. doi: 10.3390/jof9121154 (PMC10744766; doi:10.3390/jof9121154)

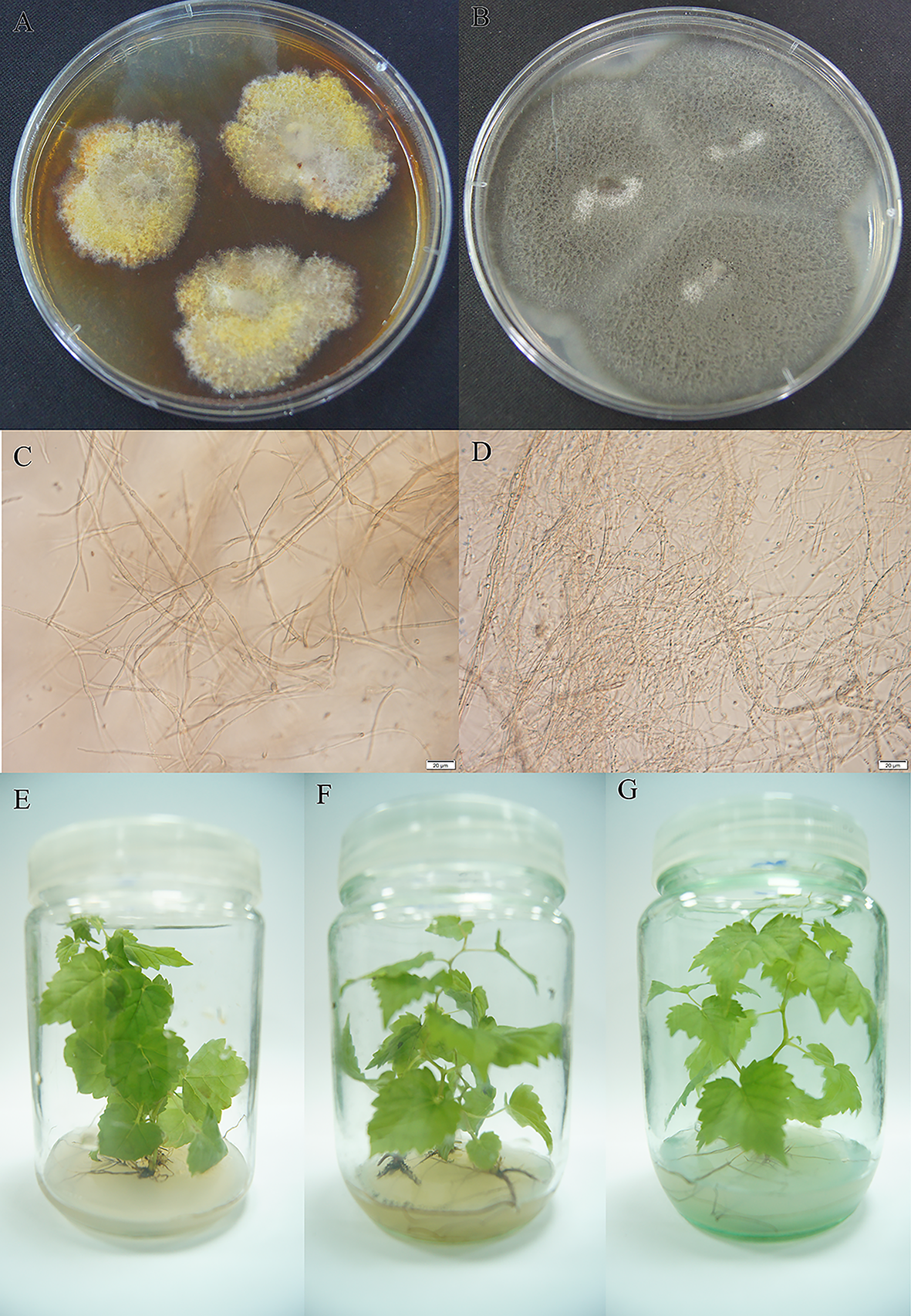

Supplement: Supplementary file 1 [file jof-09-01154-s001.zip › Figure S1.tif]

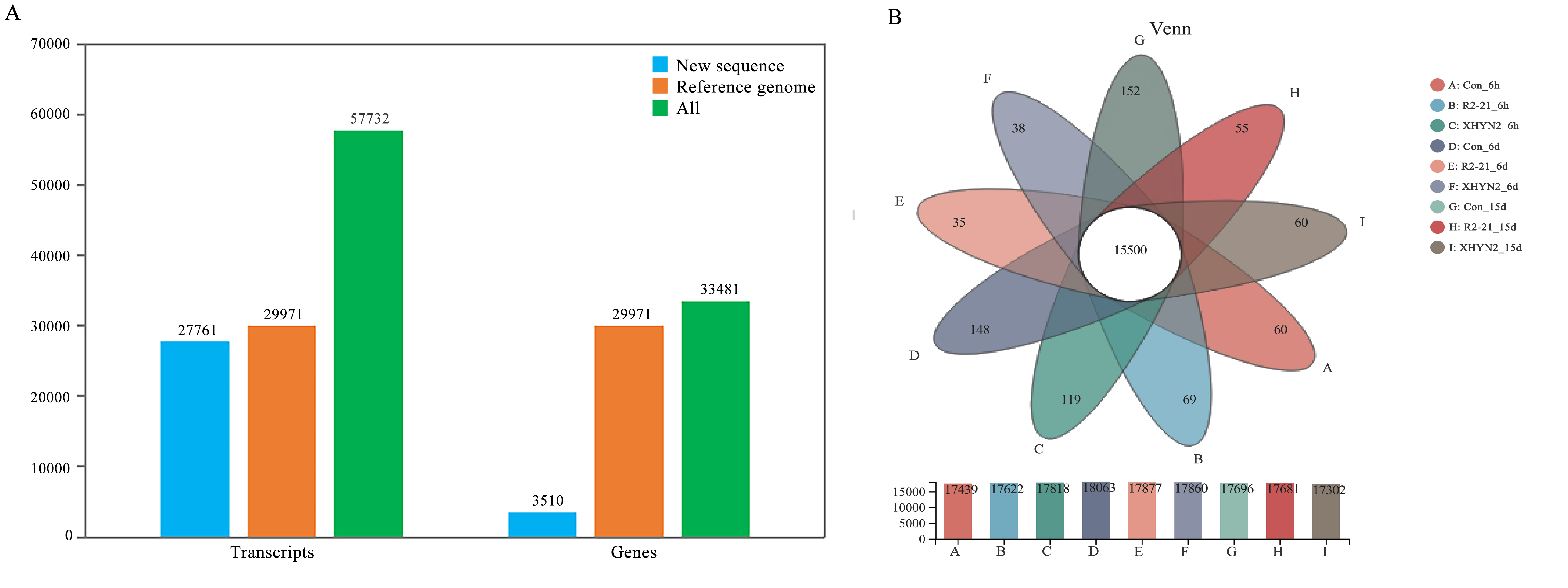

Supplement: Supplementary file 1 [file jof-09-01154-s001.zip › Figure S2.tif]

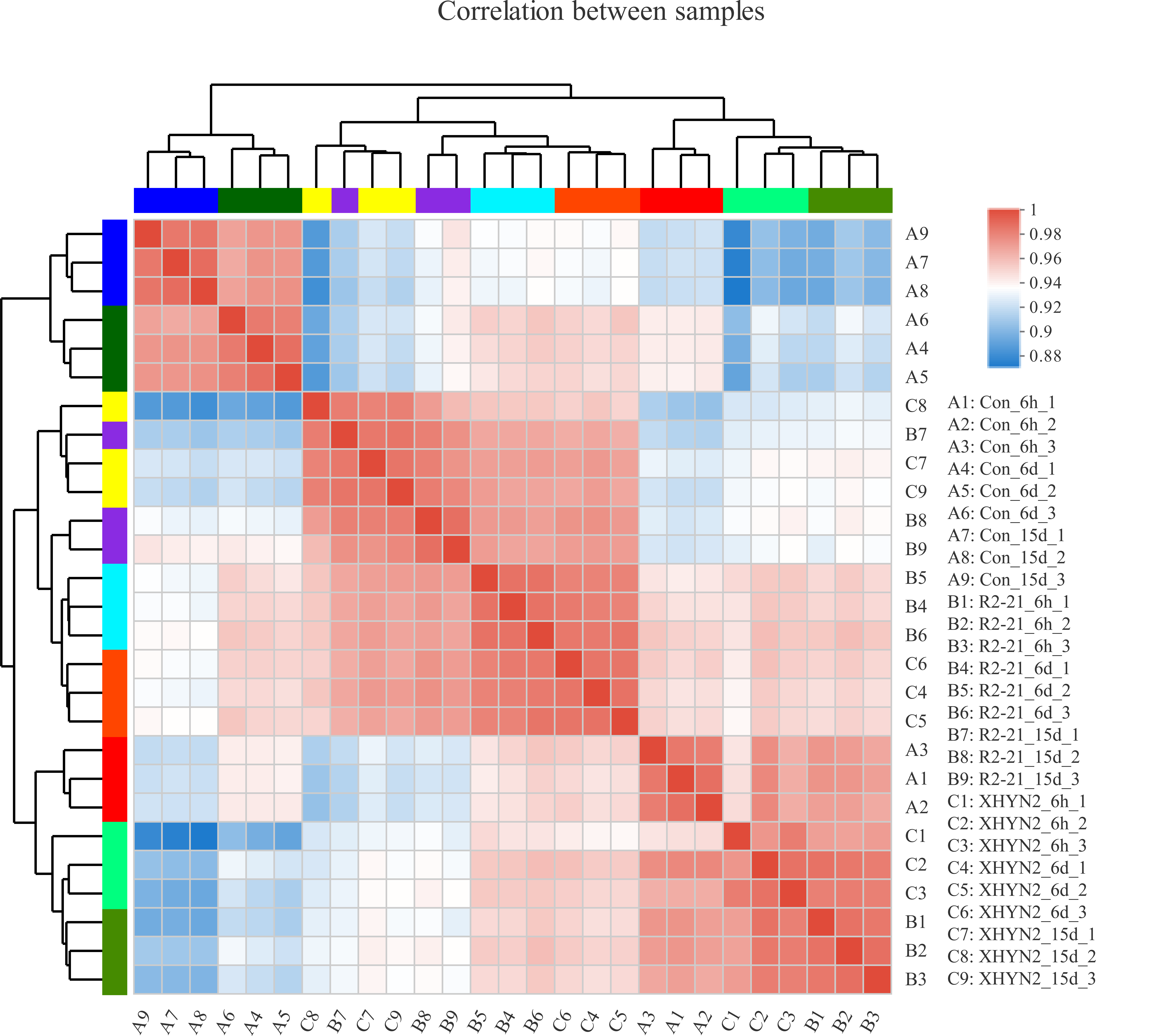

Supplement: Supplementary file 1 [file jof-09-01154-s001.zip › Figure S3.tif]

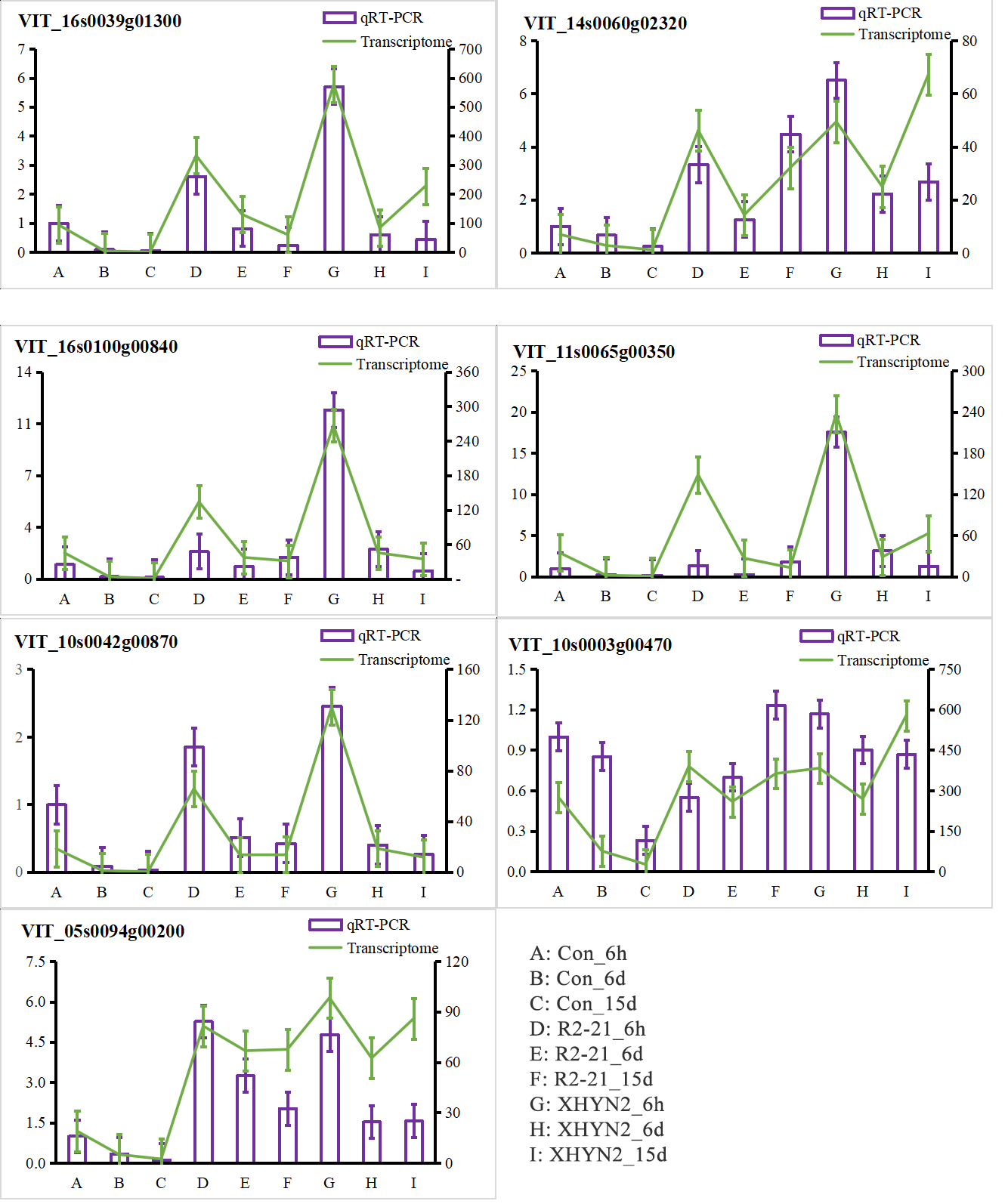

Supplement: Supplementary file 1 [file jof-09-01154-s001.zip › Figure S4.tif]

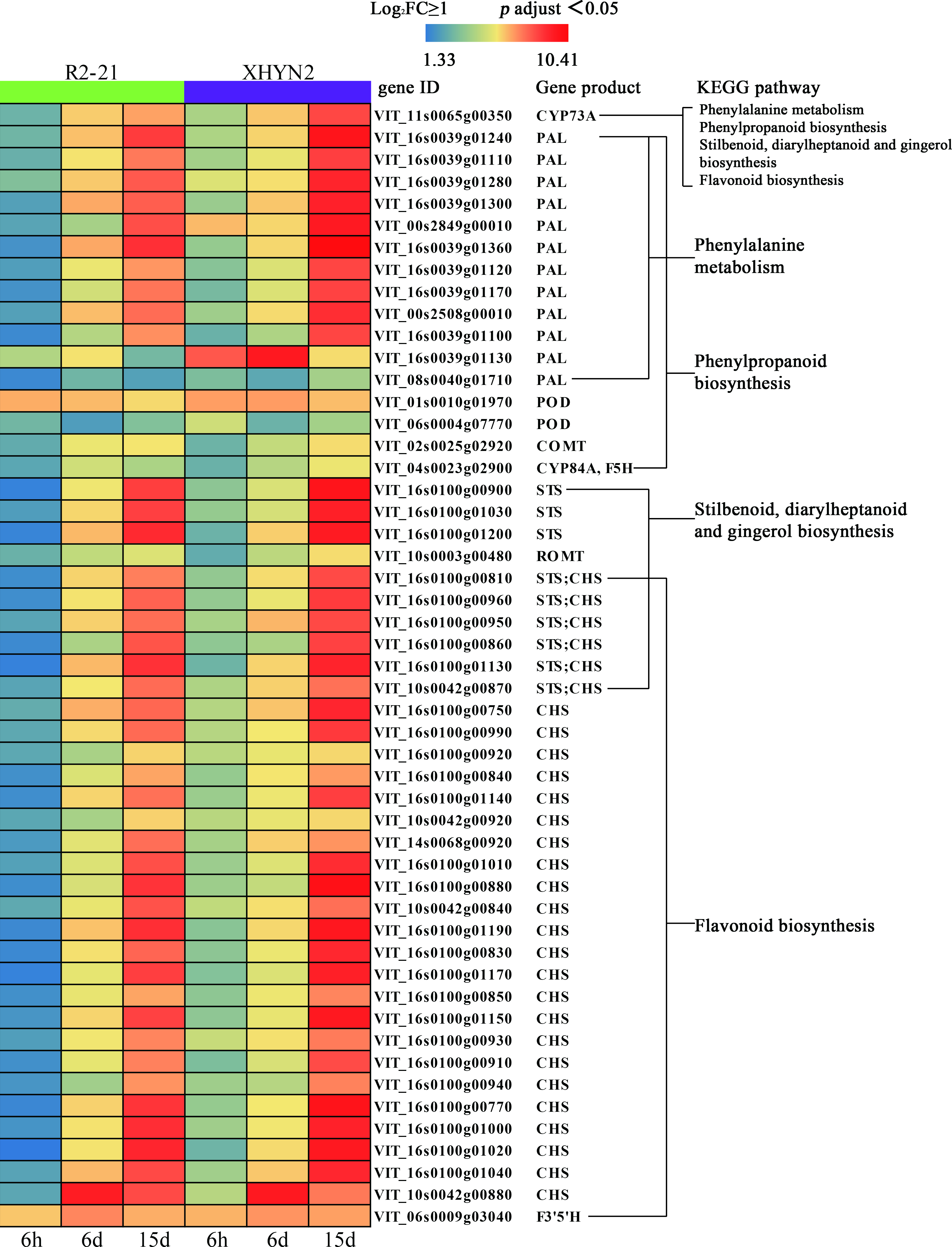

Supplement: Supplementary file 1 [file jof-09-01154-s001.zip › Figure S6.tif]

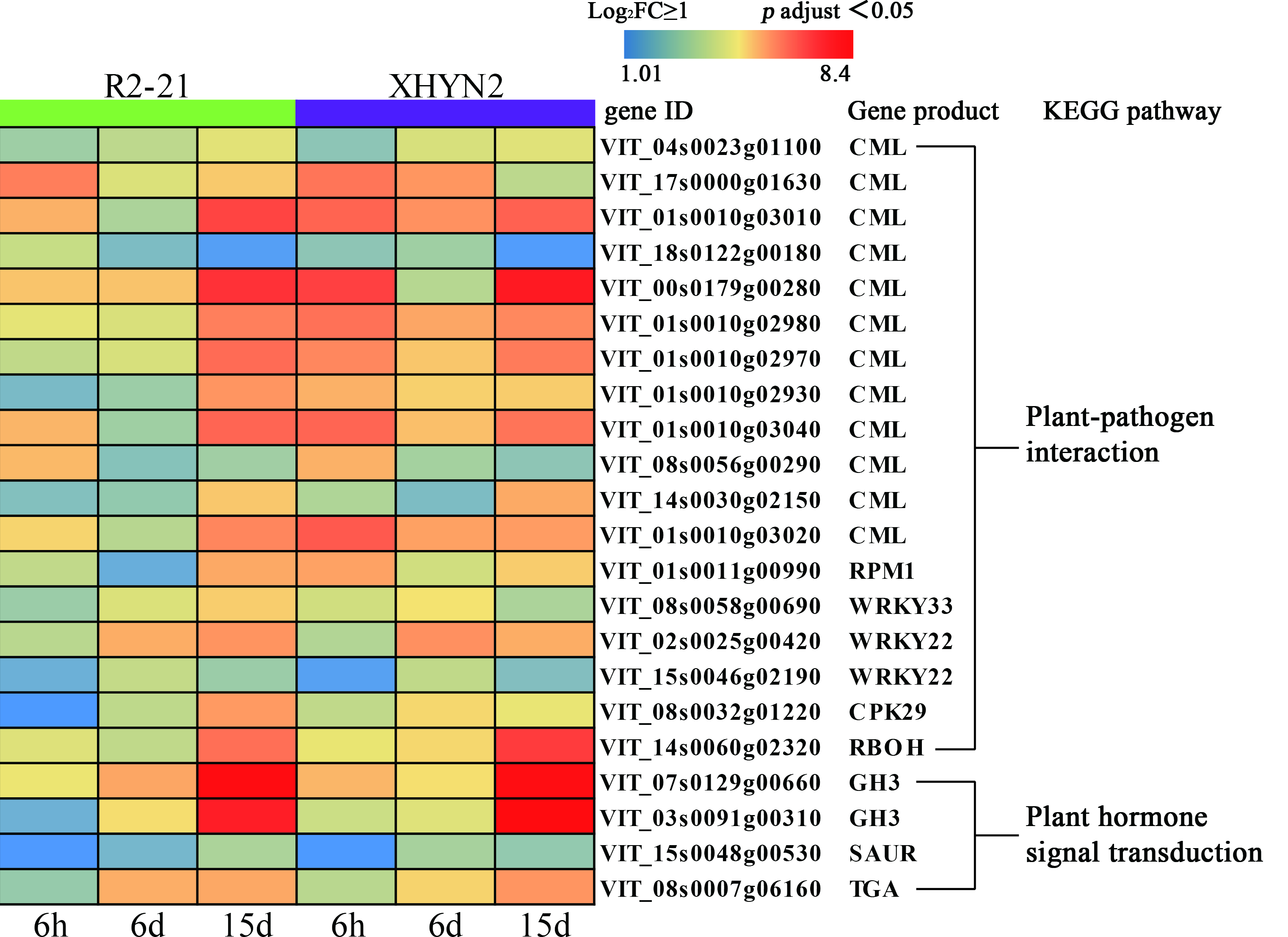

Supplement: Supplementary file 1 [file jof-09-01154-s001.zip › Figure S7.tif]
